# Promyelocytic leukemia nuclear body-like structures can assemble in mouse oocytes

**DOI:** 10.1242/bio.059130

**Published:** 2022-06-06

**Authors:** Osamu Udagawa, Ayaka Kato-Udagawa, Seishiro Hirano

**Affiliations:** Center for Health and Environmental Risk Research, National Institute for Environmental Studies, 16-2 Onogawa, Tsukuba, Ibaraki 305-8506, Japan

**Keywords:** PML-NBs, SUMO, Oocyte, Membrane-less organelle, Proteotoxic stress, Clients

## Abstract

Promyelocytic leukemia (PML) nuclear bodies (PML-NBs), a class of membrane-less cellular organelles, participate in various biological activities. PML-NBs are known as the core-shell-type nuclear body, harboring ‘client’ proteins in their core. Although multiple membrane-less organelles work in the oocyte nucleus, PML-NBs have been predicted to be absent from oocytes. Here, we show that some well-known PML clients (but not endogenous PML) co-localized with small ubiquitin-related modifier (SUMO) protein in the nucleolus and peri-centromeric heterochromatin of maturing oocytes. In oocytes devoid of PML-NBs, endogenous PML protein localized in the vicinity of chromatin. During and after meiotic resumption, PML co-localized with SUMO gathering around chromosomes. To examine the benefit of the PML-NB-free intranuclear milieu in oocytes, we deliberately assembled PML-NBs by microinjecting human PML-encoding plasmids into oocytes. Under conditions of limited SUMO availability, assembled PML-NBs tended to cluster. Upon proteotoxic stress, SUMO delocalized from peri-centromeric heterochromatin and co-localized with SC35 (a marker of nuclear speckles)-positive large compartments, which was disturbed by pre-assembled PML-NBs. These observations suggest that the PML-NB-free intranuclear environment helps reserve SUMO for emergent responses by redirecting the flux of SUMO otherwise needed to maintain PML-NB dynamics.

## Abstract (precis)

**Summary:** PML-NB-free intranuclear environment in the oocyte helps reserve SUMO for emergent responses by redirecting the flux of SUMO otherwise needed to maintain PML-NB dynamics.

## INTRODUCTION

Promyelocytic leukemia (PML) nuclear bodies (PML-NBs) are membrane-less organelles that are formed by phase separation and are present in the nuclei of mammalian interphase cells. PML-NBs consist of a shell with the ‘scaffold’ PML protein; the shell surrounds an inner core containing over 100 ‘client’ proteins. Death domain-associated protein (DAXX) and α thalassemia/mental retardation syndrome X-linked protein (ATRX) are representative client proteins in PML-NBs. These two proteins are histone chaperones that act as chromatin remodelers (Lallemand-Breitenbach and de Thé, 2018). The membrane-less property of PML-NBs facilitates the dynamic flux and interactions of PML-NB client molecules, which have been shown to be involved in a number of biological processes, including viral infection (Chelbi-Alix et al., 1995; Puvion-Dutilleul et al., 1995; Everett and Chelbi-Alix, 2007), DNA damage response (Louria-Hayon et al., 2003; Bernardi et al., 2004; Bøe et al., 2006), senescence (Ferbeyre et al., 2000; Pearson et al., 2000), and telomere recombination (Draskovic et al., 2009; Flynn et al., 2015).

Small ubiquitin-related modifier (SUMO) is a posttranslational modifier that regulates multiple cellular processes (Sahin et al., 2022). The K65, K160 and K490 residues of human PML protein are sites of SUMO conjugation (Lallemand-Breitenbach et al., 2008). Five of the six nuclear isoforms of PML (with the exception of human PMLVI) have a SUMO interaction motif (SIM) (Cappadocia et al., 2015). Via the SIM, PML non-covalently interacts with SUMO or SUMO-conjugated PML clients that also often contain SIMs. Ubc9, the only SUMO-conjugating enzyme identified thus far, also has both a site for SUMO conjugation and a SIM-like motif (Sahin et al., 2014). Therefore, SUMO can be a portion of both ‘scaffold’ and ‘client’, behaving like a matchmaker between scaffold PML and PML clients.

A decrease in the intrinsic solubility of a key molecule promotes its phase separation and formation of the membrane-less organelle (Banani et al., 2017). Arsenic directly or indirectly oligomerizes PML, inducing a sharp decline in the solubility of PML (Müller et al., 1998; Hirano et al., 2015; Udagawa and Hirano, 2022). Ubc9 is recruited to PML-NBs upon arsenic exposure. While Ubc9-driven SUMO conjugation to scaffold and clients can make PML-NBs interactive, SUMOylated PML protein is ubiquitinated by the SUMO-dependent ubiquitin E3-ligase RNF4 and then degraded via the ubiquitin-proteasome system (Lallemand-Breitenbach et al., 2008). The PML-retinoic acid receptor α fusion protein, the t(15;17) gene translocation product, disorganizes PML-NBs in the pathogenesis of acute promyelocytic leukemia (APL) (de Thé et al., 1991; Kakizuka et al., 1991). The RNF4-dependent elimination of the fusion protein is also the basis for chemotherapeutic property of arsenite.

Homozygous *Pml*^−/−^ mice exhibit leucopenia (Wang et al., 1998) and compromised innate defense (Lunardi et al., 2011). In contrast, the phenotypes of the *Pml*^−/−^ mice during reproduction and the significance of PML in the meiosis of germ cells have not been as thoroughly studied, to the best of our knowledge, likely because of the normal fecundity of *Pml*^−/−^ mice (Wang et al., 1998). PML has been demonstrated to be dispensable for embryonic development, with *Pml*^−/−^ embryos exhibiting increased resistance to acute oxidative stress compared with wild-type embryos (Niwa-Kawakita et al., 2017). These findings suggest that PML or PML-NBs play an accessory role in embryonic development. Although *Pml* mRNA is present in unfertilized mouse oocytes (Ebrahimian et al., 2010; Cho et al., 2011), the appearance of PML-NBs has not been reported, raising the possibility that *Pml* mRNA may be dormant (i.e. transcripts are stable and left untranslated). Alternatively, although PML-NBs are ubiquitously distributed in adult organisms (Goddard et al., 1995; Bernardi and Pandolfi, 2003), PML protein may not be phase-separated in a manner sufficient to form PML-NBs in oocytes.

As oocyte growth proceeds, membrane-less organelles dynamically change their characteristics and their fates. For instance, processing bodies disappear early in oocyte growth, with some components transiently forming subcortical aggregates (storage compartments for maternal mRNAs) (Flemr et al., 2010). Decreased transcription in oocytes results in a reduced number of enlarged nuclear speckles, which appear to retain unspliced pre-mRNAs (Ihara et al., 2008). The nucleolus loses its tripartite sub-compartmentation upon gradual shutdown of rRNA synthesis, forming nucleolus-like body surrounded by heterochromatin in the nuclei (Bouniol-Baly et al., 1999). These active fluxes of membrane-less organelles spatiotemporally regulate maternal RNA metabolism during oocyte growth.

Although the oocyte nucleus is an active region for membrane-less organelles, why oocytes are devoid of specific organelles such as PML-NBs and Cajal bodies remains unclear (Fulka and Aoki, 2016). Therefore, in this study, we investigated the significance of PML-NB-free intranuclear milieu of oocytes by characterizing deliberately assembled PML-NBs and analyzing their outcome in the mouse oocyte.

## RESULTS

### PML-NBs are not formed during the development of oocytes

**Fig. 1.**
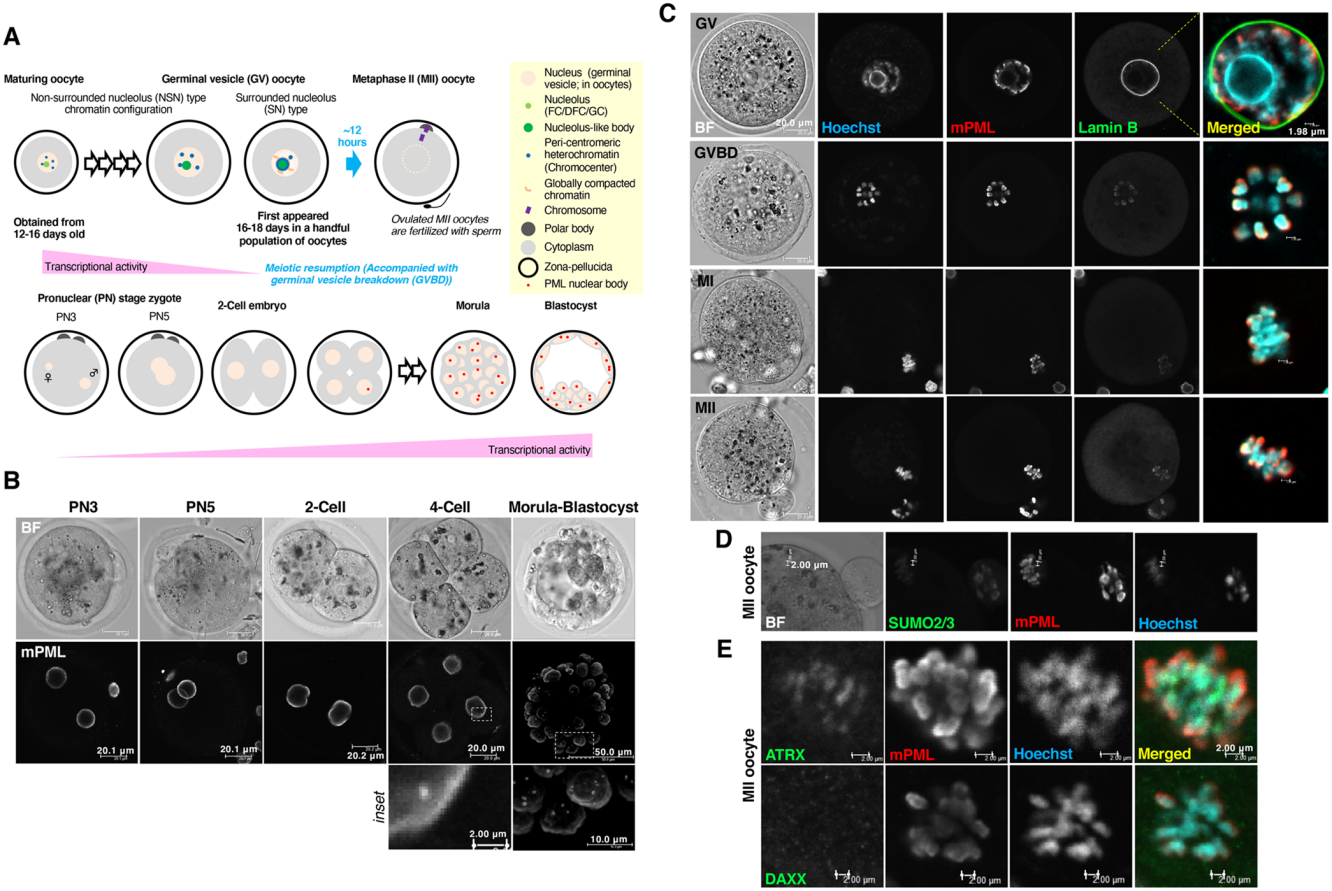
**Promyelocytic leukemia (PML) nuclear bodies (PML-NBs) are not formed during the oocyte development.** (A) Maturing oocytes with maximum transcriptional activity can be collected from approximately postnatal day 14 (De La Fuente, 2006). Like somatic cells, the nucleus of maturing oocyte includes actively transcribing, three-compartmented nucleoli (Fulka et al., 2020). These include the fibrillar center (FC, for rDNA transcription), the dense fibrillar component (DFC, for rRNA modification and processing), and the granular component (GC, for ribosome assembly). Following global transcriptional repression, oocytes undergo chromatin reorganization around the nucleolus to acquire the full competence to prepare for fertilization. The status of the full-sized oocytes remains arrested at meiotic prophase with a large nucleus, a structure that is designated the germinal vesicle (GV). The GV oocyte stage can be subdivided into the non-surrounded nucleolus (NSN) stage and the surrounded nucleolus (SN) stage based on the chromatin configuration corresponding to maturity. SN-type GV oocytes first appear at postnatal day 16–18 in a handful population of oocytes. The population increases in proportion with mouse age (Bouniol-Baly et al., 1999; Inoue et al., 2007). The SN stage is characterized by a transcriptionally inactive heterochromatin rim (i.e. with heterochromatin surrounding the nucleolus-like body). Mouse oocytes undergo growth in follicles until these cells are ready for hormone-dependent ovulation. In response to hormone signaling, fully grown GV oocytes resume meiotic division characterized by GV breakdown and subsequent polar body extrusion before fertilization with sperm. This stage is called metaphase II (MII)-stage (Racki and Richter, 2006). Pronuclear (PN) stages after insemination can be categorized based on the distance between the male and female pronuclei as follows: PN3, smaller pronuclei that are distributed distantly; PN5, bigger pronuclei that overlap. Around PN3–5 stage, the transcriptional activity of zygote/embryo begins to reactivate through to the blastocyst stage. During the development of preimplantation embryos, PML nuclear bodies appear in the nucleus of blastomeres. (B) Representative images of endogenous PML protein in zygotes and embryos at the preimplantation stages. The image of the pronuclear stage 5 (PN5) zygote was reconstructed as a z-stack image. BF, bright-field images of the zygotes and the embryos. (C) Subcellular localization of endogenous PML protein in oocytes during and after meiotic resumption. GV oocytes were cultured to obtain MII oocytes. Breakdown of the nuclear membrane was verified by staining with anti-lamin B antibody (green). PML was visualized by staining with anti-mouse PML (mPML, red) antibody. Scale bars: 20 μm. (D) Representative image of cytoplasm around the meiotic chromosome in the MII oocyte obtained by the culture of GV oocytes for 17 h. Endogenous PML protein was visualized by staining with anti-mPML (red) antibody. The oocyte was further stained with Alexa 488-conjugated anti-SUMO2/3 antibody. Vertical scale bars: 2.00 μm. (E) Representative enlarged image of cytoplasm around the meiotic chromosome in the MII oocyte obtained by the culture of GV oocytes for 15.5 h. Oocytes were stained with anti-mPML (red) and anti-ATRX or DAXX (green) antibodies. Scale bars: 2.00 μm.

To begin with, PML-NBs were seen in 98.1% of the 162 examined bone marrow cells used as a positive control (Fig. S1B). During the development of preimplantation embryos, PML-NBs were detected in the nucleus of each blastomere in the morula to early blastocyst stage (Fig. 1A,B; Fig. S1A). PML protein co-localized with its clients (Fig. S1A) and was slightly enriched beneath the nuclear membrane (Fig. S1A). While PML-NBs were seen faintly in 15.6% of 32 blastomeres examined at the 4-cell embryo stage, PML protein was not detected as NBs in the pronuclei of fertilized zygotes or the nuclei of 2-cell embryos. No NBs were observed in the nucleoplasm among the 35 examined 2-cell embryos (Fig. 1B).

To determine the location of PML protein in oocytes heading for fertilization, germinal vesicle (GV) oocytes were collected from ovaries and cultured *in vitro*. PML protein localized in the vicinity of chromatin, wrapping the nucleolus-like body as the growth of GV oocytes proceeds (Fig. 1A,C; Fig. S3B). The peri-chromosomal distribution of PML protein was observed in oocytes in the GV breakdown, MI, and MII stages, corresponding to during and after the resumption of meiosis (Fig. 1A,C,D). The localization of ATRX in MII oocytes was chromosomal, in contrast to the localization of PML and SUMO (Fig. 1D,E). Enrichment of DAXX was not observed at the chromosomal region (Fig. 1E). Immuno-EM analysis (using anti-mouse PML primary antibody and gold-conjugated secondary antibody) revealed that gold particles (indicative of PML-positive staining) were localized peri-chromosomally as amorphous-like structures (Fig. S1C). Immunofluorescence analysis of PML, in combination with staining for a chromosomal passenger complex marker and a kinetochore marker revealed that PML likely localizes on the chromosome arms (Fig. S1D,E). In contrast to mitotic assemblies of PML proteins (MAPPs), which are tethered to early endosomes in metaphase-stage somatic cells (Dellaire et al., 2006), PML in metaphase oocytes did not localize at early endosomes (Fig. S1F).

These results indicate that PML protein was not detected as NBs in oocytes.

### PML-NBs potentially affect the response of SUMO upon exposure to proteotoxic stress

**Fig. 2.**
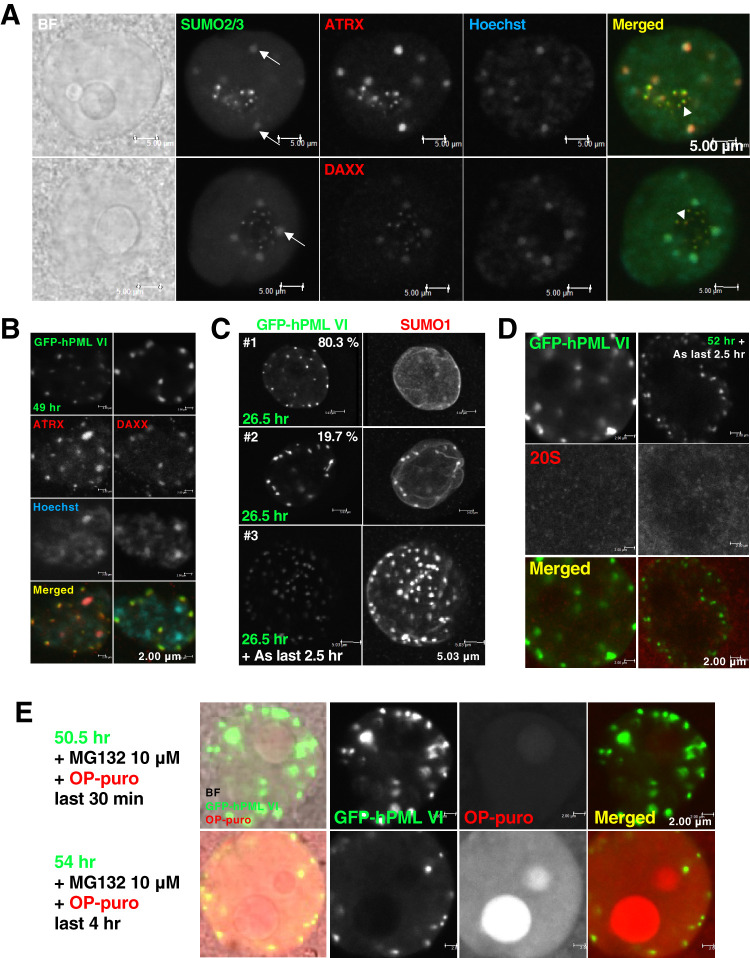
**Characterization of nuclear bodies (NBs) deliberately assembled in the nuclei of oocytes.** (A) Representative images of the nuclei of maturing oocytes left untreated for 2 h. Oocytes were stained with Alexa 488-conjugated anti-SUMO2/3 antibody and anti-ATRX or DAXX antibodies. BF, bright-field images. Scale bars: 5.00 μm. (B) Representative images of exogenously formed GFP-hPMLVI-NBs and of representative PML clients in the nuclei of maturing oocytes. Actively transcribing maturing oocytes were used to obtain a higher efficiency of plasmid expression. Oocytes were visualized by immunofluorescent staining with anti-ATRX or DAXX (red) antibodies. Scale bars: 2.00 μm. (C) Representative image of the nucleus of maturing oocytes injected with a plasmid encoding GFP-hPMLVI and cultured for 24 h, then either treated with 3 μM arsenite (As) or left untreated for another 2.5 h. Oocytes were stained with anti-human PML (green) and anti-SUMO1 (red) antibodies. (#1) While GFP-hPMLVI-NBs were formed, a lower degree of SUMO sequestration was observed. (#2) GFP-hPMLVI-NBs were fully formed, and SUMO was well sequestered. Scale bars: 5.03 μm. (#3, z-stack) Sequestration of SUMO, even by the faintly visible GFP-hPMLVI-NBs. Scale bars: 5.03 μm. (D) Representative image of the nucleus of a maturing oocytes injected with the plasmid encoding GFP-hPMLVI and cultured for 49.5 h, then either treated with 3 μM arsenite or left untreated for another 2.5 h. Oocytes were stained with anti-proteasome 20S alpha 1+2+3+5+6+7 (20S) (red) antibody. Scale bars: 2.00 μm. (E) An examination of whether GFP-hPMLVI-NBs act as overflow compartments for misfolded proteins. Representative image of the nucleus of maturing oocytes injected with the plasmid encoding GFP-hPMLVI and cultured for 50 h before labeling of newly synthesized aberrant polypeptides with an analog of puromycin (OP-puro, 20 μM) (red) combined with treatment of proteasome inhibitor (MG132, 10 μM) for 30 min or 4 h. Scale bars: 2.00 μm.

To test whether the PML-NB-free intranuclear environment has any benefits for oocytes, we first attempted the assembly of NBs in the nuclei of GV oocytes. For simplicity, we injected a plasmid that encodes GFP-hPMLVI, the transcript variant 5 of human PML that lacks a SIM, to avoid non-covalent interaction with SUMO or the SUMOylated moieties of modified clients that often also contain SIMs. Even with a low success rate of expression (<10%), presumably because of the transcriptional quiescence of GV oocytes, injection of this construct resulted in the formation of NBs in the nuclei of GV oocytes (Fig. S2A). In these oocytes, the sequestration of endogenous PML clients into GFP-hPMLVI-NBs was observed (Fig. S2B). A higher efficiency of plasmid expression (reaching >70%) was obtained in actively transcribing maturing oocytes. Maturing oocytes obtained from approximately postnatal day 12–16 (see Fig. 1A) are meiotically incompetent (De La Fuente, 2006). SUMO localized primarily in the nucleoplasm, with several depositions at sites of condensed DNA (i.e. peri-centromeric heterochromatin) in maturing oocytes (Fig. 2A). A portion of the SUMO signal was detected and finely co-localized with those of PML clients in a dot-like appearance in the nucleolus (Fig. 2A). However, endogenous PML was not detected in the nucleolus (Fig. S2C). The dot-like signal, seen in 92.2% of 34 maturing oocytes, remained even after the nucleolar structures were disrupted with actinomycin D, a transcriptional inhibitor (Fig. S2D). The sequestration of PML clients into GFP-hPMLVI-NBs was observed (Fig. 2B,C). Approximately 19.7% of 66 maturing oocytes exhibited evident NBs. With an additional day of plasmid expression, the percentage increased to 68% of 25 maturing oocytes tested (Fig. 2C). In the presence of arsenite, even the faintly visible nascent GFP-hPMLVI-NBs co-localized with SUMO (Fig. 2C).

We next assessed the effects of assembly of PML-NB on SUMOylation-triggered PML catabolism. A previous study reported that SUMO-dependent catabolic machineries are recruited to SUMOylated PML-NBs exposed to arsenite (Lallemand-Breitenbach et al., 2008). However, the signal of 20S proteasome was not detected at GFP-hPMLVI-NBs (Fig. 2D). Like the nucleolus, PML-NBs also have been reported to act as overflow compartments for misfolded proteins in the nuclei of somatic cells (Uozumi et al., 2016; Mediani et al., 2019). Since the oocyte is PML-NB-free, the incapacity for dealing with the proteinous wastes could be a disadvantage for oocytes. In the presence of the proteasome inhibitor MG132, the aberrant polypeptides labeled with an analog of puromycin (OP-puro) accumulated only in the nucleolus but not in GFP-hPMLVI-NBs (Fig. 2E). Similarly, in morula-blastocyst stage embryos under proteotoxic stress, OP-puro-labeled aberrant polypeptides did not accumulate at endogenous PML-NBs (Fig. S2E).

**Fig. 3.**
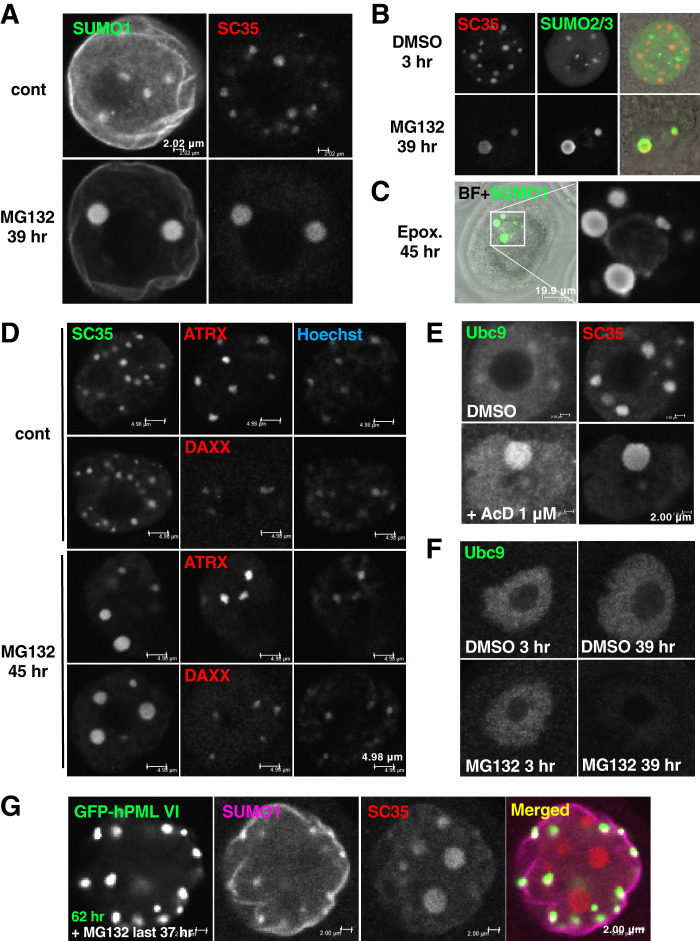
**PML-NBs potentially affect the efficiency of the response of SUMO upon exposure to proteotoxic stress.** (A–C) Prolonged exposure to proteasome inhibitors. Representative images of the nuclei of maturing oocytes subjected to treatment with 10 μM MG132 for 39 h (A,B), or with 1 μM epoxomicin for 45 h (C, Epox.). Oocytes were stained with anti-splicing component, 35 kDa (SC35) (red) and anti-SUMO1 (green) (A) or Alexa 488-conjugated anti-SUMO2/3 (green) (B), or with anti-SUMO1 (green) only (C, z-stack). BF, bright-field image of the oocyte. (D) The response of representative PML clients to proteasome inhibition. Representative images of the nuclei of maturing oocytes left untreated (cont) or treated with 10 μM MG132 for 45 h. Oocytes were stained with anti-SC35 (green) and anti-ATRX or DAXX (red) antibodies. Scale bars: 4.98 μm. (E) The response of SC35-positive compartments to transcriptional inhibition. Representative image of the nucleus of maturing oocytes treated with dimethyl sulfoxide (DMSO, vehicle control) or 1 μM actinomycin D (AcD) for 16 h. Oocytes were stained with anti-SC35 (red) and anti-Ubc9 (green) antibodies. Scale bars: 2.00 μm. (F) Representative images of the nuclei of maturing oocytes treated with DMSO or 10 μM MG132 for the indicated time. Oocytes were stained with anti-Ubc9 antibody. (G) An examination of whether exogenously assembled PML-NBs alter the response of SUMO to proteotoxic stress. Representative image of the nucleus of maturing oocytes injected with a plasmid encoding GFP-hPMLVI and cultured for 25 h followed by treatment with 10 μM MG132 for 37 h. Oocytes were stained with anti-SUMO1 (magenta) and anti-SC35 (red) antibodies. Scale bars: 2.00 μm.

SC35 is a well-defined marker of nuclear speckles (Ihara et al., 2008). In proteotoxic assays, we found that prolonged exposure of maturing oocytes with proteasome inhibitors resulted in co-localization of SUMO signal with enlarged SC35-positive compartments (Fig. 3A–C). While the SUMO signal disappeared from peri-centromeric heterochromatin upon prolonged proteasomal inhibition (Fig. 3A), PML clients maintained localization at the peri-centromeric heterochromatin (Fig. 3D). Since SC35-positive compartments are enlarged upon transcriptional repression (Ihara et al., 2008), we assessed whether transcriptional repression is involved in the formation of enlarged SC35-positive compartments upon prolonged proteasomal inhibition. While Ubc9 accumulated in large SC35-positive nuclear speckles upon transcriptional repression as reported in the literature (Ihara et al., 2008) (Fig. 3E), no specific accumulation of Ubc9 was observed upon treatment of MG132 (Fig. 3F). These results suggested that the response of SUMO co-localization with enlarged SC35-positive compartments upon prolonged proteasomal inhibition is distinct from the response to transcriptional repression. Accordingly, in maturing oocytes, after assembly of PML-NBs by the injection of the GFP-hPMLVI-encoding plasmid and exposure to prolonged proteasomal inhibition, SUMO was not sequestered to enlarged SC35-positive compartments but to GFP-hPMLVI-NBs (Fig. 3G). Considering the data indicating that assembled PML-NBs in oocytes are not involved in the regulation of misfolded proteins upon proteotoxic stress (Fig. 2D,E), these findings collectively suggest that the assembly of PML-NBs indirectly affects the SUMO-related response to proteotoxic stress.

To evaluate another type of proteotoxic stress, we tested heat shock (HS; incubation at 42°C for 2 h). While maturing oocytes were vulnerable, GV oocytes were slightly resilient to HS. In NSN-stage GV oocytes, SUMO localized primarily in the nucleoplasm, with several depositions at peri-centromeric heterochromatin (Fig. S3A), similarly as described in maturing oocytes. Upon HS, SUMO exhibited co-localization with SC35-positive compartments (Fig. S3A). In SN-stage GV oocytes, SUMO was highly enriched along the heterochromatin rim (Fig. S3A), while SC35-positive compartments disappeared. Upon HS, SUMO co-localized with enlarged SC35-positive compartments (Fig. S3A). In HS, the enlarged SC35-positive compartments did not contain endogenous PML (Fig. S3B) and the signals of ATRX disappeared (Fig. S3C). The signal of ATRX was weakened upon MG132 treatment, only remaining at subsets of heterochromatin (Fig. S3C). We attempted to examine the effect of deliberate assembly of PML-NBs. Although mRNA injection is a highly effective method for driving gene expression in GV oocytes, we were unable to assemble PML-NBs in GV oocytes by injecting *hPMLVI* mRNA unless the recipient oocytes were exposed to additional stimuli (Fig. S3D). The enlarged SC35-positive compartments were no longer observed in embryos exposed to HS at the 4-cell stage (Fig. S3E), nor at the morula-blastocyst stage (Fig. S3F), when endogenous PML-NBs emerge.

### The dynamics of PML-NBs in the nucleoplasm demand SUMO

**Fig. 4.**
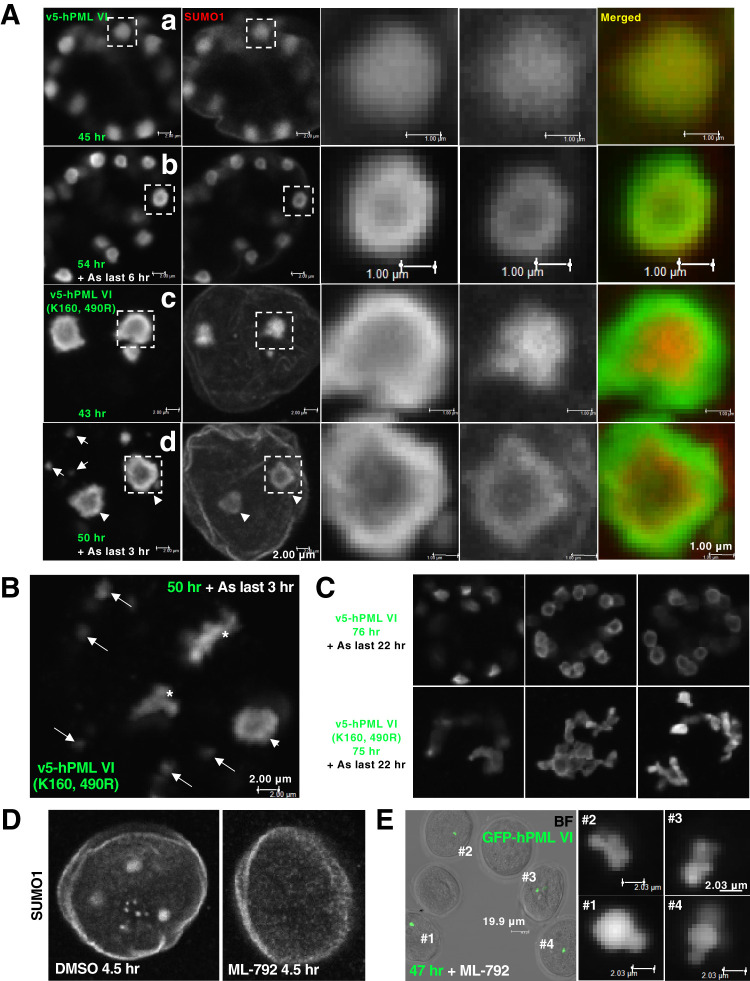
**The dynamics of PML-NBs in the nucleoplasm demand SUMO.** (A) Representative image of the nucleus of maturing oocytes injected with plasmid v5-hPMLVI (encoding wild-type human PMLVI) and cultured for 45 h (A-a), with plasmid v5-hPMLVI and cultured for 48 h followed by treatment with 3 μM arsenite (As) for 6 h (A-b), with plasmid v5-hPMLVI (K160, 490R) (encoding a human PML VI that has limited avidity for SUMO) and cultured for 43 h (A-c, z-stack), or with plasmid v5-hPMLVI (K160, 490R) and cultured for 47 h followed by treatment with 3 μM arsenite for 3 h (A-d, z-stack). Oocytes were stained with anti-human PML (green) and SUMO1 (red) antibodies. Arrows, v5-hPMLVI (K160, 490R)-NBs newly formed upon arsenite treatment; arrowheads, enlarged v5-hPMLVI (K160, 490R)-NBs. Scale bars: 2.00 μm. Enlarged images of marked areas are in the insets. Scale bars: 1.00 μm. (B) Representative z-stack image of the nucleus of maturing oocytes injected with plasmid v5-hPMLVI (K160, 490R) and cultured for 47 h followed by treatment with 3 μM arsenite for 3 h. Oocytes were stained with anti-human PML (green) antibody. Asterisks, v5-hPMLVI (K160, 490R)-NBs, newly formed upon arsenite treatment, that clustered frequently. Scale bars: 2.00 μm. (C) Representative images of the nuclei of maturing oocytes injected with plasmid v5-hPMLVI or v5-hPMLVI (K160, 490R) subjected to prolonged treatment with 3 μM arsenite. Oocytes were stained with anti-human PML (green) antibody. (D) Representative z-stack image of the nucleus of maturing oocytes treated with dimethyl sulfoxide (DMSO, vehicle control) or with 20 μM ML-792, an inhibitor of a SUMO-activating enzyme, for 4.5 h. Oocytes were stained with anti-SUMO1 antibody. (E) Representative enlarged z-stack images of GFP-hPMLVI-NBs assembled in maturing oocytes. Oocytes were injected with a plasmid encoding GFP-hPMLVI and cultured in the presence of 20 μM ML-792 for 47 h. Oocytes were stained with anti-human PML (green) antibody. BF, bright-field image of the oocytes. Scale bars: 19.9 μm (insets #1 to #4, 2.03 μm).

To analyze the role of SUMO availability for assembled PML-NBs in detail, maturing oocytes were first manipulated to limit the availability of SUMO with which PML could interact. Specifically, we injected oocytes with a construct [v5-hPMLVI (K160, 490R)] that encodes the SIM-free PML isoform containing mutations of the SUMO conjugation sites, thereby decreasing the protein's avidity for SUMO and the SIM moiety of SUMOylated clients (Fig. S4A,B). We found that oocytes injected with plasmid v5-hPMLVI (K160, 490R) exhibited decreased numbers of nucleolar-sized PML structures [2.77±0.24 (mean±s.e.m.), across 48 oocytes] (Fig. 4A-c, Fig. S4C) compared with the higher numbers in oocytes injected with the wild-type PMLVI-encoding plasmid [20.45±1.29 (mean±s.e.m.), across 20 oocytes] (Fig. 4A-a). In the v5-hPMLVI (K160, 490R)-injected oocytes, SUMO accumulated but not at the shell of the PML structures; SUMO protein appeared to be isolated within the PML-negative inner core (Fig. 4A-c). We next promoted the availability of SUMO with which PML could interact by applying arsenite. Arsenite efficiently promotes SUMOylation of PML. Notably, the SUMOylation-deficient mutant of PML remained biochemically responsive to arsenite. Similar to findings from a previous report (Lallemand-Breitenbach et al., 2001), the v5-hPMLVI (K160, 490R)-encoded protein exhibited a sharp decline in solubility comparable with that of wild type (Fig. S4B). In oocytes injected with the wild-type plasmid, arsenite treatment altered the spatial relationship between PML and SUMO from co-localization to spherical co-layering (Fig. 4A-b). In line with this observation, arsenite treatment rescued the aberrant SUMO localization in the v5-hPMLVI (K160, 490R)-injected oocytes (Fig. 4A-d). Of note, we observed the nascent formation of small-dot structures upon arsenite treatment in the v5-hPMLVI (K160, 490R)-injected oocytes (Fig. 4A-d, B; 44.2% of 43 oocytes exhibited this phenotype). We found that these structures became heavily clustered as the arsenite exposure prolonged (Fig. 4B,C).

Next, to understand the clustering of the nascent v5-hPMLVI (K160, 490R)-NBs, we treated maturing oocytes with ML-792 (an inhibitor of a SUMO-activating enzyme; He et al., 2017) to limit the nucleoplasmic availability of SUMO. The nuclei of maturing oocytes treated with ML-792 did not show general staining for anti-SUMO1 antibody and exhibited staining only at the nuclear membrane (Fig. 4D). Maturing oocytes injected with the GFP-hPMLVI-encoding plasmid in the absence of ML-792 exhibited discrete NBs [60.62±6.03 (mean±s.e.m.), across 30 oocytes] (Fig. S4D), and those cultured in the presence of ML-792 exhibited a decreased number (1.96±0.18; 27 oocytes cultured for a shorter period of 22 h were quantified) that clustered together (Fig. 4E). Furthermore, we examined the sequestration of endogenous PML or its clients into v5-hPMLVI- or v5-hPMLVI (K160, 490R)-NBs. While finding endogenous PML sequestrated into GFP-hPMLVI-NBs was difficult, the sequestration of PML into v5-hPMLVI- or v5-hPMLVI (K160, 490R)-NBs was undetectable. The localizations of endogenous PML and assembled PML-NBs were often mutually exclusive (Fig. S4D). Sequestration of PML clients into v5-hPMLVI-NBs was observed. Compared with DAXX (Fig. S4F), ATRX was prone to keep its peri-centromeric heterochromatin localization even when being sequestrated (Fig. S4E), consistent with the result obtained with GFP-hPMLVI-NBs (Fig. 2B). The sequestration of ATRX into arsenite-dependent doughnut-like v5-hPMLVI-NBs was observed in 31.6% of 19 doughnut-phenotype oocytes (Fig. S4E). While the sequestration of ATRX into v5-hPMLVI (K160, 490R)-NBs was undetectable even in the presence of arsenite (Fig. S4E), a faint signal of DAXX could be detectable (Fig. S4F) in a manner similar to that of SUMO. These results suggest that assembled PML-NBs maintain the dynamics at the cost of SUMO availability in the oocyte.

## DISCUSSION

At least three SUMO isoforms are expressed ubiquitously in mammals. SUMO2 is the major isoform expressed in embryogenesis, and *Sumo2*-deficient mice die at approximately embryonic day 10.5 (Wang et al., 2014). In contrast, neither SUMO1 nor SUMO3 is essential for embryogenesis. There are three distinct steps in SUMOylation. Ubc9, which is required for the second reaction, is also indispensable for embryogenesis. Like embryos lacking SUMO2, *Ubc9*-deficient embryos die during embryogenesis, at approximately embryonic day 3.5–7.5, that is, just after implantation (Nacerddine et al., 2005). Mice harboring an oocyte-specific knockout of the Ubc9-encoding gene (*Ube2i-cKO*) show infertility with complete failure of oocytes to extrude the polar bodies (but no aberrancy in spindle morphology) (Rodriguez et al., 2019), supporting the notion that SUMOylation is critical for regulation of molecules involved in the MI–MII transition (Wang et al., 2010; Yuan et al., 2014; Ding et al., 2018). Although the role of endogenous PML (mPML) remains to be clarified, we found that mPML gradually began to co-localize with SUMO during and after the meiotic resumption (Fig. 1D; Fig. S3B). In the *Ube2i-cKO* study, Rodriguez et al. used a *Gdf-9 Cre* promoter as a driver (so that *Ube2i* deletion begins at approximately postnatal day 3); the observed dysfunction of gonadotropin-primed GV oocytes collected from 3-week-old mice implies that the SUMOylation pathway is required for processes that begin before the GV stage as previously suggested (Ihara et al., 2008). While the substrates of SUMOylation are largely unknown, these findings suggest the significance of SUMO availability in the nucleoplasm of maturing oocytes. In the present study, we showed that well-defined PML clients (but not mPML) co-localized with SUMO not only in the nucleolus but also in peri-centromeric heterochromatin of maturing oocytes. The nucleolus is a site of active ribosomal biogenesis. The role of PML clients in the nucleolus of oocytes is unknown. HS-dependent deposition of DAXX, a PML client, at peri-centromeric heterochromatin was detected, while DAXX was sequestrated in PML-NBs in normal conditions in cancer cell lines (Morozov et al., 2012). Intriguingly, one report showed that differentiation-dependent loss of PML expression in myogenic cells redirects DAXX from PML-NBs to chromatin (Salsman et al., 2017). The differentiation-dependent loss of PML expression was also reported in neuronal differentiation (Aoto et al., 2006; Regad et al., 2009). In oocytes devoid of PML-NBs, PML protein is expressed and localized in the vicinity of chromatin (Fig. 1C; Fig. S3B). In oocytes, PML clients remain even upon proteotoxic stress (Fig. 3D), while SUMO delocalized from peri-centromeric heterochromatin and co-localized with SC35-positive large structures, which was disturbed by pre-assembled PML-NBs (Fig. 3G). The response of SUMO2/3 against stress including HS is more pronounced than that of SUMO1 (Saitoh and Hinchey, 2000; Seifert et al., 2015). Although biochemical analysis is needed, SUMO1 also responded to the proteotoxic stress in oocytes (Fig. 3A–C). Given the histone chaperone activity of DAXX/ATRX complex, these data collectively suggest that the PML-NB-free environment may help maintain chromatin architecture in oocytes. This is consistent with the observation that the localizations of almost all assembled PML-NBs and that of mPML were mutually exclusive (Fig. S4D).

**Fig. 5.**
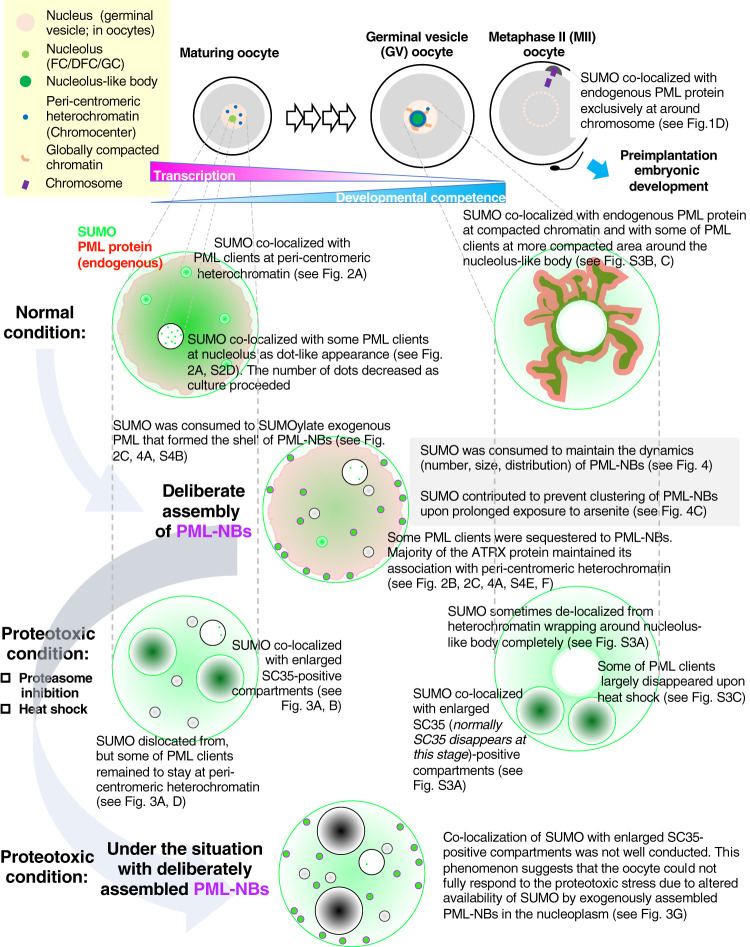
**Potential significance of PML-NB-free intranuclear environment of oocytes as exemplified by the outcomes of deliberate PML-NBs assembly.** As oocytes acquire the developmental competence, chromatin status and their configurations are subjected to drastic alterations that correlate with transcriptional quiescence. Co-localization of SUMO (green) with endogenous PML protein (red) was gradually evident upon meiotic resumption followed by fertilization (normal condition, right side). However, during the maturation of oocytes, well-defined PML clients but not endogenous PML protein co-localized with SUMO not only in the nucleolus but also in peri-centromeric heterochromatin (normal condition, left side). While the selected PML clients remained to stay at peri-centromeric heterochromatin, SUMO committed to the response against proteotoxic stress. SUMO delocalized from peri-centromeric heterochromatin and co-localized with SC35-positive large structures (black) (proteotoxic condition, left side). Microinjection experiments for deliberate assembly of PML-NBs (purple, forming the ‘shell’) revealed that SUMO contributed to the regulation of dynamics as well as prevention of clustering of PML-NBs in the nucleoplasm of oocytes. Under the situation with deliberately assembled PML-NBs, the emergent response of SUMO was not adequately conducted, suggesting that the PML-NB-free intranuclear environment helps reserve SUMO for emergent responses by redirecting the flux of SUMO otherwise needed to maintain PML-NB dynamics.

A recent study employing polymer physics proposed a model for the organization of paraspeckles, another core-shell-type nuclear body. The report showed that parts of the building domains of the scaffold molecules (long non-coding RNAs) are mutually repulsive when positioned at the surface of paraspeckle, which accordingly determines the dynamics (size, number, and distribution) of the nuclear body; however, these repulsive domains are not required for the assembly of paraspeckle itself (Yamazaki et al., 2021). Notably, SUMOylation is dispensable for the formation of PML-NBs (Sahin et al., 2014). As shown in the wild-type v5-hPMLVI-injected oocytes (Fig. 4A), arsenite treatment induces rapid polymerization of SUMO chains on insolubilized PML at the shell (Fig. S4B), such that the structure forms a spherical co-layer (Fig. 4A-b). PML-NBs show a core-shell ultrastructure at the electron microscopic level (Lallemand-Breitenbach et al., 2001), and therefore the arsenite-dependent formation of doughnut-like v5-hPMLVI-NBs appears topologically ideal for the sequestration of PML clients. However, such structures often failed to sequestrate PML clients (Fig. S4E,F), suggesting that the observed doughnut-like topology of the shell might simply be a physicochemical adaptation (e.g. minimization of surface tension) to the nucleoplasmic properties of oocytes. The crooked-shaped shells of v5-hPMLVI (K160, 490R)-NBs (Fig. 4A-c), whose avidity for SUMO is limited, may indicate that the availability of SUMO in the oocyte nucleoplasm is one of the determinants of the surface property of PML-NBs facing the nucleoplasm. The exfoliated-appearance of SUMO molecules in the inner core of v5-hPMLVI (K160, 490R)-NBs may be from the reactivity of SIM-containing clients like DAXX to SUMO is elicited (Fig. S4F). Arsenite-dependent co-layer appearance of SUMO molecules in v5-hPMLVI (K160, 490R)-NBs (Fig. 4A-d) is probably from the residual potency for SUMOylation via K65 as described. The clustering potential of v5-hPMLVI (K160, 490R)-NBs upon prolonged arsenite exposure (Fig. 4C) suggests that if PML-NBs are present in the nucleus of oocytes, SUMO would be highly consumed to prevent irregular clustering so as to maintain the homogenous nucleoplasmic distribution of PML-NBs, even in unhealthy conditions. As depicted with summarizing schemes of the present study (Fig. 5), the PML-NB-free intranuclear milieu of oocytes thus may reflect the significance of the reserve of SUMO available for emergent responses (e.g. proteotoxic condition).

Oocytes and embryos are special cells that often undergo drastic temperature changes in freezing/thawing during fertility treatment. The younger the stage of oocytes, the greater the number of the oocytes; using younger oocytes may be of benefit to patients with less ovulated oocytes. However, further improvement of culture technique will be required to use younger oocytes. Understanding the basic properties of the stress response of oocytes may contribute to the development of conditions that keep oocytes healthy during maturation to be fertilizable with sperm.

## MATERIALS AND METHODS

### Chemicals, reagents, and antibodies

Sodium arsenite (NaAsO_2_), Triton X-100, 3-isobutyl-1-methylxanthine (IBMX), and K-modified simplex optimized medium (KSOM) were purchased from Sigma-Aldrich (St. Louis, MO, USA). Paraformaldehyde (PFA), dimethyl sulfoxide (DMSO), bovine serum albumin (BSA), actinomycin D (AcD), and kanamycin were purchased from WAKO (Osaka, Japan). Pregnant mare serum gonadotropin (PMSG) and human chorionic gonadotropin (hCG) were purchased from Sigma-Aldrich and ASKA Pharmaceutical (Tokyo, Japan). Modified human tubular fluid (mHTF) medium was purchased from Kyudo (Saga, Japan). Carbobenzoxy-L-leucyl-L leucyl-L-leucinal (MG132) was purchased from Calbiochem (San Diego, CA, USA). Epoxomicin was purchased from Enzo Life Sciences (Farmingdale, NY, USA). α-minimum essential media (MEM) medium, penicillin/streptomycin, and 4-(2-hydroxyethyl)-1-piperazineethanesulfonic acid (HEPES) were purchased from Gibco/Thermo Fisher Scientific (Grand Island, NY, USA). Lipofectamine LTX-Plus reagents, TOP10 competent cells, NuPAGE 4-12% Bis-Tris gels, lithium dodecyl sulfate (LDS) sample buffer, Bolt™ WB systems, and MagicMark™XP size standards were purchased from Invitrogen/Thermo Fisher Scientific (Carlsbad, CA, USA). Mineral oil was purchased from Nacalai Tesque (Kyoto, Japan). ML-792 was purchased from MedKoo Biosciences (Morrisville, NC, USA). Hoechst dye was purchased from Dojin Chemical (Kumamoto, Japan). DNase was purchased from Ambion/Thermo Fisher Scientific (Carlsbad, CA, USA). Radioimmunoprecipitation (RIPA) lysis solutions (containing 0.1% sodium dodecyl sulfate), protease inhibitor cocktail, phenylmethylsulfonyl fluoride (PMSF), and sodium orthovanadate (SOV) were purchased from Santa Cruz Biotechnology (Santa Cruz, CA, USA). KOD-FX and KOD-plus Mutagenesis Kits were purchased from TOYOBO (Osaka, Japan). Fetal bovine serum (FBS) was purchased from Biowest (Nuaille, France). ECL^TM^ Prime was purchased from GE Healthcare (Buckinghamshire, UK). The following antibodies were used in this study: anti-α thalassemia/mental retardation syndrome X-linked protein (ATRX), anti-death domain-associated protein (DAXX), anti-human PML (sc-966), Alexa 488-conjugated anti-SUMO2/3, anti-lamin B, horseradish peroxidase (HRP) -conjugated goat anti-mouse or -rabbit immunoglobulin G (IgG) (Santa Cruz Biotechnology), anti-human PML (A301-167A: Bethyl, Montgomery, TX, USA), anti-mouse PML (#05-718: Millipore), anti-SUMO1 (#4940), anti-survivin, anti-early endosome antigen 1 (EEA1) (Cell Signaling Technology, Danvers, MA, USA), anti-SUMO2/3 (#M114-3: MBL, Nagoya, Japan), calcinosis Raynaud's phenomenon esophageal dysmotility, sclerodactyly and telangiectasia (CREST) human autoantibody (HCT-0100: Immuno Vision, Springdale, USA), anti-fibrillarin (ab5821), anti-SUMO-conjugating enzyme (Ubc9), anti-proteasome 20*S* alpha 1+2+3+5+6+7 (20S) (Abcam, Cambridge, UK), anti-splicing component, 35 kDa (SC35) (S4045: Sigma-Aldrich), and Alexa 488-, 594-, or 647-conjugated secondary antibodies (Molecular Probes/Thermo Fisher Scientific). The #M114-3 clone of anti-SUMO2/3 antibody also was used to generate additional Alexa 488-conjugated anti-SUMO2/3 antibody, which was labeled using an Alexa Fluor^TM^ 488 Antibody Labeling Kit (Molecular Probes/Thermo Fisher Scientific, Eugene, OR, USA).

### Collection and culture of oocytes, zygotes, and embryos

All animal procedures and protocols were in accordance with the ‘Guidelines for the Care and Use of Laboratory Animals’ (June 2021 edition) and were approved by the Animal Care and Use Committee of the National Institute for Environmental Studies (Approval No. 21-002). C57BL/6J mice were purchased from CLEA-Japan (Kawasaki, Japan). The animals were housed under a 12-hr/12-hr light/dark cycle with free access to food and water. Unless otherwise mentioned, oocytes were cultured in medium for *in vitro* maturation [basal medium: α-MEM medium supplemented with penicillin/streptomycin (100 units/ml and 100 μg/ml, respectively) and heat-inactivated FBS (10%)]. Actively transcribed and meiotically incompetent oocytes collected from postnatal days 12–16 are referred to as ‘maturing oocytes’ in the present study. Except for the oocytes depicted in Fig. S1D–F (which represent samples collected directly from adult ovaries), Metaphase I (MI) and Metaphase II (MII)-stage oocytes were obtained by *in vitro* culture as follows. For collection of fully grown GV oocytes, follicles on the ovarian surface were mechanically ruptured with a pair of forceps. GV oocytes initially were isolated in basal medium supplemented with HEPES (12.5 mM) and IBMX (0.1 mM); IBMX is a phosphodiesterase inhibitor that inhibits/controls spontaneous meiotic resumption. After IBMX was removed by washing with basal medium, oocytes were incubated for 4 h to allow meiotic resumption to proceed. Development of oocytes was further verified by observation of the extrusion of the first polar body (Pb1; a marker of MII-stage oocytes that have the potential to be fertilized with sperm) after an additional incubation of up to 16 h. We regarded Pb1-free oocytes with no GV as MI-stage oocytes. To obtain embryos, female mice were initially primed by intraperitoneal injection of PMSG followed 48 h later by injection with hCG. Superovulated oocytes were collected from the oviducts of euthanized mice by gently teasing apart of the ampulla with a 21-gauge needle (TERUMO, Tokyo, Japan) to release cumulus-oocyte complexes in mHTF medium; these oocytes then were inseminated with pre-capacitated sperm. After fertilization, 2-cell embryos were cultured further until the indicated embryonic stages or time points using KSOM medium. Cultures were performed in a drop of medium (30–80 µL) under a mineral oil overlay at 37°C in a humidified atmosphere of 5% CO_2_ (APM-30D; ASTEC, Fukuoka, Japan), except for heat shock experiments, which were performed at 42°C in ambient air (FMC-1000; EYELA, Tokyo, Japan).

### Western blot analysis for PML and SUMO in CHO-K1 cells

Using Lipofectamine LTX-Plus reagents, CHO-K1 cells were transiently transfected with plasmids harboring *PmlVI* (human PML transcript variant 5, NM_033244) or a cDNA encoding a SUMOylation-deficient version of the protein. The former construct, which we designated v5-hPMLVI, was obtained from OriGene (Rockville, MD, USA). The latter construct, which we designated v5-hPMLVI (K160, 490R), encodes a mutant version of the same protein in which the nucleic acid sequences encoding the K160 and K490 amino acid residues were altered to encode Arg residues (by mutation using the KOD-plus Mutagenesis Kit according to the manufacturer's instructions). The transfected cells were washed with phosphate-buffered saline (PBS, pH 7.1–7.7) and then lysed on ice with 100 µl of RIPA lysis buffer pre-mixed with protease inhibitor cocktail (1:100, vol/vol), PMSF (a serine protease inhibitor; 2 mM), and SOV (a phosphatase inhibitor; 1 mM). The lysate was centrifuged at 9000×***g*** for 5 min at 4°C and the resulting supernatant was designated as the RIPA-soluble fraction. The pellet was washed again with PBS and then treated with DNase (40 U/ml) to reduce the viscosity. Pellets were ruptured by ultrasonication at 4°C for 30 min using a Bioruptor™ (UCD-250, H-amplitude, repeating 15-s sonication at 45-s intervals; Cosmobio, Tokyo, Japan) followed by a 1-h incubation at 25°C with intermittent vortexing using a thermomixer (Eppendorf, Wesseling-Berzdorf, Germany). Aliquots were designated as the RIPA-insoluble fraction and subsequently mixed with LDS sample buffer and boiled at 95°C for 3 min before being stocked frozen at −30°C. Before loading onto gels, samples again were boiled at 95°C for 10 min. Proteins in the samples were resolved by LDS-polyacrylamide gel electrophoresis (LDS-PAGE) and electroblotted to membranes; the membranes were blocked and then probed with primary antibodies overnight at 4°C before hybridization with secondary antibodies for 1 h at room temperature. Signals obtained with ECL^TM^ Prime Chemiluminescent HRP Substrate were detected with an FAS1100 (TOYOBO).

### Plasmid or mRNA microinjection

The Emerald Green Fluorescent Protein (EmGFP) -encoding region from Vivid Colors™ cDNA pcDNA™6.2/N-EmGFP-DEST Vector (Thermo Fisher Scientific) was placed upstream of the coding sequence of *PmlVI* (human PML transcript variant 5, NM_033244) as described previously (Hirano et al., 2018). The fusion protein encoded by the resulting construct was designated GFP-hPMLVI. Each plasmid, including v5-hPMLVI and v5-hPMLVI (K160, 490R), was diluted to a concentration of 5 ng/µl with Milli-Q water. mRNA preparation was conducted by BEX (Tokyo, Japan) by *in vitro* transcription from v5-hPMLVI or v5-hPMLVI (K160, 490R) using a HiScribe™ T7 ARCA mRNA Kit with tailing (New England Biolabs, Ipswich, MA, USA) and a MEGAclear™ Transcription Clean-Up Kit (Thermo Fisher Scientific). Each mRNA was diluted to a concentration of 25 or 100 ng/µl with RNase free water (Jena Bioscience, Jena, Germany). Each solution was loaded into a DNA injection pipette (TIP-DNA [LIC-OD1], NAKA Medical, Tokyo, Japan), and the pipettes were placed in an IM-9B microinjector (Narishige, Tokyo, Japan). MN-4 and MMO-202ND manipulators (Narishige) were adapted to an IX70 inverted microscope (Olympus, Tokyo, Japan) via NO-PIX-4-P (Narishige). Plasmid or mRNA solutions were injected into each oocyte nucleus or cytoplasm (respectively) until adequate swelling of the nuclear or plasma membrane (respectively) was observed. Injection was conducted in the basal medium supplemented with HEPES (12.5 mM) and IBMX (0.1 mM). Injected oocytes were washed and cultured without HEPES during the interval indicated for each experiment.

### Immunofluorescent staining

Except for the live imaging of GV oocytes in Fig. S2A, oocytes, zygotes, and embryos at each indicated stage were fixed with 4% phosphate-buffered PFA (pH 7.0–7.4) at room temperature. Cells were permeabilized with 0.5% Triton X-100 in PBS, blocked with 5% BSA-PBS, and then stained with the indicated primary antibodies in 1% BSA-PBS. Cells were visualized with Alexa-conjugated secondary antibodies; Hoechst dye was included to stain DNA. Cells were placed in small drops (4 µl each) of 1% BSA-PBS, covered with mineral oil, in a glass-bottomed 35-mm Petri dish (AGC techno glass, Shizuoka, Japan). Images were captured by confocal microscopy (Leica TCS-SP5; Leica, Solms, Germany).

For the detection of nascently translated polypeptides, oocytes or embryos were cultured with OP-puro (obtained as part of a Click-iT^®^ Plus OPP Protein Synthesis Assay Kit; Molecular Probes/Thermo Fisher Scientific) at a concentration of 20 µM for the indicated time periods, followed by Click reaction according to the manufacturer's instructions. Images were captured by confocal microscopy.

As a reference for PML-NB staining, bone marrow from euthanized male C57BL/6J mice was exposed by removing both ends of the femur; bone marrow cells then were obtained by flushing the marrow with PBS using a 25-gauge needle (TERUMO). The resulting cells were immediately cytocentrifuged onto slide glass and air-dried. The cell preparations were used for immunostaining for visualization of endogenous PML-NBs. Images were captured by fluorescence microscopy (ECLIPSE 80i; Nikon, Tokyo, Japan).

### Electron microscopic (EM) analysis

GV oocytes were cultured for 20 h. Metaphase oocytes were fixed in 4% PFA, 0.2% glutaraldehyde, and 0.5% tannic acid in 0.1 M cacodylate buffer, pH 7.4, for 100 min at room temperature. The fixed oocytes then were washed with 0.1 M cacodylate buffer. Further procedures were conducted by Tokai Electron Microscopy, Inc. (Nagoya, Japan). After dehydration, the sample was embedded in resin (LR White; London Resin, Berkshire, UK). The polymerized resin was ultra-thin sectioned at thicknesses of 80 nm, and the sections were mounted on nickel grids. The grids were incubated with the primary antibody (anti-mouse PML, #05–718) in 1% BSA-PBS overnight at 4°C. The grid-mounted sections subsequently were incubated for 1 h at room temperature with the secondary antibody conjugated to 20-nm gold particles (EMGMHL20; BBI Solutions, Crumlin, UK). Grids then were placed in 2% glutaraldehyde in 0.1 M cacodylate buffer. Subsequently, the grids were dried and stained with 2% uranyl acetate for 15 min, and then placed in lead staining solution (Sigma-Aldrich) at room temperature for 3 min. The grids were observed by transmission electron microscopy (JEM-1400Plus; JEOL, Tokyo, Japan).

### Data analysis

Where indicated, data are presented as means with the standard error of the mean (s.e.m.).

## Supplementary Material

Supplementary information

## References

[BIO059130C1] Aoto, T., Saitoh, N., Ichimura, T., Niwa, H. and Nakao, M. (2006). Nuclear and chromatin reorganization in the MHC-Oct3/4 locus at developmental phases of embryonic stem cell differentiation. *Dev. Biol.* 298, 354-367. 10.1016/j.ydbio.2006.04.450 16950240

[BIO059130C2] Banani, S. F., Lee, H. O., Hyman, A. A. and Rosen, M. K. (2017). Biomolecular condensates: organizers of cellular biochemistry. *Nat. Rev. Mol. Cell Biol.* 18, 285-298. 10.1038/nrm.2017.7 28225081 PMC7434221

[BIO059130C3] Bernardi, R. and Pandolfi, P. P. (2003). Role of PML and the PML-nuclear body in the control of programmed cell death. *Oncogene* 22, 9048-9057. 10.1038/sj.onc.1207106 14663483

[BIO059130C4] Bernardi, R., Scaglioni, P. P., Bergmann, S., Horn, H. F., Vousden, K. H. and Pandolfi, P. P. (2004). PML regulates p53 stability by sequestering Mdm2 to the nucleolus. *Nat. Cell Biol.* 6, 665-672. 10.1038/ncb1147 15195100

[BIO059130C5] Bøe, S. O., Haave, M., Jul-Larsen, Å., Grudic, A., Bjerkvig, R. and Lønning, P. E. (2006). Promyelocytic leukemia nuclear bodies are predetermined processing sites for damaged DNA. *J. Cell Sci.* 119, 3284-3295. 10.1242/jcs.03068 16868026

[BIO059130C6] Bouniol-Baly, C., Hamraoui, L., Guibert, J., Beaujean, N., Szöllösi, M. S. and Debey, P. (1999). Differential transcriptional activity associated with chromatin configuration in fully grown mouse germinal vesicle oocytes. *Biol. Reprod.* 60, 580-587. 10.1095/biolreprod60.3.580 10026102

[BIO059130C7] Cappadocia, L., Mascle, X. H., Bourdeau, V., Tremblay-Belzile, S., Chaker-Margot, M., Lussier-Price, M., Wada, J., Sakaguchi, K., Aubry, M., Ferbeyre, G. et al. (2015). Structural and functional characterization of the phosphorylation-dependent interaction between PML and SUMO1. *Structure* 23, 126-138. 10.1016/j.str.2014.10.015 25497731

[BIO059130C8] Chelbi-Alix, M., Pelicano, L., Quignon, F., Koken, M., Venturini, L., Stadler, M., Pavlovic, J., Degos, L. and de Thé, H. (1995). Induction of the PML protein by interferons in normal and APL cells. *Leukemia* 9, 2027-2033. 8609713

[BIO059130C9] Cho, S., Park, J. S. and Kang, Y.-K. (2011). Dual functions of histone-lysine N-methyltransferase Setdb1 protein at promyelocytic leukemia-nuclear body (PML-NB) maintaining PML-NB structure and regulating the expression of its associated genes. *J. Biol. Chem.* 286, 41115-41124. 10.1074/jbc.M111.248534 21921037 PMC3220519

[BIO059130C10] De La Fuente, R. (2006). Chromatin modifications in the germinal vesicle (GV) of mammalian oocytes. *Dev. Biol.* 292, 1-12. 10.1016/j.ydbio.2006.01.008 16466710

[BIO059130C11] de Thé, H., Lavau, C., Marchio, A., Chomienne, C., Degos, L. and Dejean, A. (1991). The PML-RARα fusion mRNA generated by the t (15; 17) translocation in acute promyelocytic leukemia encodes a functionally altered RAR. *Cell* 66, 675-684. 10.1016/0092-8674(91)90113-D 1652369

[BIO059130C12] Dellaire, G., Eskiw, C. H., Dehghani, H., Ching, R. W. and Bazett-Jones, D. P. (2006). Mitotic accumulations of PML protein contribute to the re-establishment of PML nuclear bodies in G1. *J. Cell Sci.* 119, 1034-1042. 10.1242/jcs.02817 16492707

[BIO059130C13] Ding, Y., Kaido, M., Llano, E., Pendas, A. M. and Kitajima, T. S. (2018). The post-anaphase SUMO pathway ensures the maintenance of centromeric cohesion through meiosis I-II transition in mammalian oocytes. *Curr. Biol.* 28, 1661-1669.e4. 10.1016/j.cub.2018.04.019 29754905

[BIO059130C14] Draskovic, I., Arnoult, N., Steiner, V., Bacchetti, S., Lomonte, P. and Londoño-Vallejo, A. (2009). Probing PML body function in ALT cells reveals spatiotemporal requirements for telomere recombination. *Proc. Natl Acad. Sci. USA* 106, 15726-15731. 10.1073/pnas.0907689106 19717459 PMC2747187

[BIO059130C15] Ebrahimian, M., Mojtahedzadeh, M., Bazett-Jones, D. and Dehghani, H. (2010). Transcript isoforms of promyelocytic leukemia in mouse male and female gametes. *Cells Tissues Organs* 192, 374-381. 10.1159/000319466 20664248

[BIO059130C16] Everett, R. D. and Chelbi-Alix, M. K. (2007). PML and PML nuclear bodies: implications in antiviral defence. *Biochimie* 89, 819-830. 10.1016/j.biochi.2007.01.004 17343971

[BIO059130C17] Ferbeyre, G., de Stanchina, E., Querido, E., Baptiste, N., Prives, C. and Lowe, S. W. (2000). PML is induced by oncogenic ras and promotes premature senescence. *Genes Dev.* 14, 2015-2027. 10.1101/gad.14.16.2015 10950866 PMC316863

[BIO059130C18] Flemr, M., Ma, J., Schultz, R. M. and Svoboda, P. (2010). P-body loss is concomitant with formation of a messenger RNA storage domain in mouse oocytes. *Biol. Reprod.* 82, 1008-1017. 10.1095/biolreprod.109.082057 20075394 PMC2857638

[BIO059130C19] Flynn, R. L., Cox, K. E., Jeitany, M., Wakimoto, H., Bryll, A. R., Ganem, N. J., Bersani, F., Pineda, J. R., Suvà, M. L. and Benes, C. H. (2015). Alternative lengthening of telomeres renders cancer cells hypersensitive to ATR inhibitors. *Science* 347, 273-277. 10.1126/science.1257216 25593184 PMC4358324

[BIO059130C20] Fulka, H. and Aoki, F. (2016). Nucleolus precursor bodies and ribosome biogenesis in early mammalian embryos: old theories and new discoveries. *Biol. Reprod* 94, 143. 10.1095/biolreprod.115.136093 26935600

[BIO059130C21] Fulka, H., Rychtarova, J. and Loi, P. (2020). The nucleolus-like and precursor bodies of mammalian oocytes and embryos and their possible role in post-fertilization centromere remodelling. *Biochem. Soc. Trans.* 48, 581-593. 10.1042/BST20190847 32318710

[BIO059130C22] Goddard, A., Yuan, J., Fairbairn, L., Dexter, M., Borrow, J., Kozak, C. and Solomon, E. (1995). Cloning of the murine homolog of the leukemia-associated PML gene. *Mamm. Genome* 6, 732-737. 10.1007/BF00354296 8563172

[BIO059130C23] He, X., Riceberg, J., Soucy, T., Koenig, E., Minissale, J., Gallery, M., Bernard, H., Yang, X., Liao, H., Rabino, C. et al. (2017). Probing the roles of SUMOylation in cancer cell biology by using a selective SAE inhibitor. *Nat. Chem. Biol.* 13, 1164-1171. 10.1038/nchembio.2463 28892090

[BIO059130C24] Hirano, S., Tadano, M., Kobayashi, Y., Udagawa, O. and Kato, A. (2015). Solubility shift and SUMOylaltion of promyelocytic leukemia (PML) protein in response to arsenic(III) and fate of the SUMOylated PML. *Toxicol. Appl. Pharmacol.* 287, 191-201. 10.1016/j.taap.2015.05.018 26049103

[BIO059130C25] Hirano, S., Udagawa, O., Kobayashi, Y. and Kato, A. (2018). Solubility changes of promyelocytic leukemia (PML) and SUMO monomers and dynamics of PML nuclear body proteins in arsenite-treated cells. *Toxicol. Appl. Pharmacol.* 360, 150-159. 10.1016/j.taap.2018.10.001 30292834

[BIO059130C26] Ihara, M., Stein, P. and Schultz, R. M. (2008). UBE2I (UBC9), a SUMO-conjugating enzyme, localizes to nuclear speckles and stimulates transcription in mouse oocytes. *Biol. Reprod* 79, 906-913. 10.1095/biolreprod.108.070474 18703419 PMC2714998

[BIO059130C27] Inoue, A., Akiyama, T., Nagata, M. and Aoki, F. (2007). The perivitelline space-forming capacity of mouse oocytes is associated with meiotic competence. *J. Reprod. Dev.* 53, 1043-1052. 10.1262/jrd.19064 17587772

[BIO059130C28] Kakizuka, A., Miller, W., Jr, Umesono, K., Warrell, R., Jr, Frankel, S. R., Murty, V., Dmitrovsky, E. and Evans, R. (1991). Chromosomal translocation t (15; 17) in human acute promyelocytic leukemia fuses RARα with a novel putative transcription factor, PML. *Cell* 66, 663-674. 10.1016/0092-8674(91)90112-C 1652368

[BIO059130C29] Lallemand-Breitenbach, V. and de Thé, H. (2018). PML nuclear bodies: from architecture to function. *Curr. Opin. Cell Biol.* 52, 154-161. 10.1016/j.ceb.2018.03.011 29723661

[BIO059130C30] Lallemand-Breitenbach, V., Zhu, J., Puvion, F., Koken, M., Honoré, N., Doubeikovsky, A., Duprez, E., Pandolfi, P. P., Puvion, E., Freemont, P. et al. (2001). Role of promyelocytic leukemia (PML) sumolation in nuclear body formation, 11S proteasome recruitment, and As2O3-induced PML or PML/retinoic acid receptor α degradation. *J. Exp. Med.* 193, 1361-1372. 10.1084/jem.193.12.1361 11413191 PMC2193303

[BIO059130C31] Lallemand-Breitenbach, V., Jeanne, M., Benhenda, S., Nasr, R., Lei, M., Peres, L., Zhou, J., Zhu, J., Raught, B. and de Thé, H. (2008). Arsenic degrades PML or PML–RARα through a SUMO-triggered RNF4/ubiquitin-mediated pathway. *Nat. Cell Biol.* 10, 547-555. 10.1038/ncb1717 18408733

[BIO059130C32] Louria-Hayon, I., Grossman, T., Sionov, R. V., Alsheich, O., Pandolfi, P. P. and Haupt, Y. (2003). The promyelocytic leukemia protein protects p53 from Mdm2-mediated inhibition and degradation. *J. Biol. Chem.* 278, 33134-33141. 10.1074/jbc.M301264200 12810724

[BIO059130C33] Lunardi, A., Gaboli, M., Giorgio, M., Rivi, R., Bygrave, A., Antoniou, M., Drabek, D., Dzierzak, E., Fagioli, M., Salmena, L. et al. (2011). A role for PML in innate immunity. *Genes Cancer* 2, 10-19. 10.1177/1947601911402682 21779477 PMC3111006

[BIO059130C34] Mediani, L., Guillén-Boixet, J., Vinet, J., Franzmann, T. M., Bigi, I., Mateju, D., Carrà, A. D., Morelli, F. F., Tiago, T., Poser, I. et al. (2019). Defective ribosomal products challenge nuclear function by impairing nuclear condensate dynamics and immobilizing ubiquitin. *EMBO J.* 38, e101341. 10.15252/embj.2018101341 31271238 PMC6669919

[BIO059130C35] Morozov, V. M., Gavrilova, E. V., Ogryzko, V. V. and Ishov, A. M. (2012). Dualistic function of Daxx at centromeric and pericentromeric heterochromatin in normal and stress conditions. *Nucleus* 3, 276-285. 10.4161/nucl.20180 22572957 PMC3414404

[BIO059130C36] Müller, S., Miller, W. H., Jr and Dejean, A. (1998). Trivalent antimonials induce degradation of the PML-RARα oncoprotein and reorganization of the promyelocytic leukemia nuclear bodies in acute promyelocytic leukemia NB4 cells. *Blood* 92, 4308-4316. 10.1182/blood.V92.11.4308.423k36_4308_4316 9834237

[BIO059130C37] Nacerddine, K., Lehembre, F., Bhaumik, M., Artus, J., Cohen-Tannoudji, M., Babinet, C., Pandolfi, P. P. and Dejean, A. (2005). The SUMO pathway is essential for nuclear integrity and chromosome segregation in mice. *Dev. Cell* 9, 769-779. 10.1016/j.devcel.2005.10.007 16326389

[BIO059130C38] Niwa-Kawakita, M., Ferhi, O., Soilihi, H., Le Bras, M., Lallemand-Breitenbach, V. and de Thé, H. (2017). PML is a ROS sensor activating p53 upon oxidative stress. *J. Exp. Med.* 214, 3197-3206. 10.1084/jem.20160301 28931625 PMC5679165

[BIO059130C39] Pearson, M., Carbone, R., Sebastiani, C., Cioce, M., Fagioli, M., Saito, S. I., Higashimoto, Y., Appella, E., Minucci, S. and Pandolfi, P. P. (2000). PML regulates p53 acetylation and premature senescence induced by oncogenic Ras. *Nature* 406, 207-210. 10.1038/35018127 10910364

[BIO059130C40] Puvion-Dutilleul, F., Chelbi-Alix, M. K., Koken, M., Quignon, F., Puvion, E. and de Thé, H. (1995). Adenovirus infection induces rearrangements in the intranuclear distribution of the nuclear body-associated PML protein. *Exp. Cell Res.* 218, 9-16. 10.1006/excr.1995.1125 7737384

[BIO059130C41] Racki, W. J. and Richter, J. D. (2006). CPEB controls oocyte growth and follicle development in the mouse. *Development* 133, 4527-4537. 10.1242/dev.02651 17050619

[BIO059130C42] Regad, T., Bellodi, C., Nicotera, P. and Salomoni, P. (2009). The tumor suppressor Pml regulates cell fate in the developing neocortex. *Nat. Neurosci.* 12, 132-140. 10.1038/nn.2251 19136970

[BIO059130C43] Rodriguez, A., Briley, S. M., Patton, B. K., Tripurani, S. K., Rajapakshe, K., Coarfa, C., Rajkovic, A., Andrieux, A., Dejean, A. and Pangas, S. A. (2019). Loss of the E2 SUMO-conjugating enzyme Ube2i in oocytes during ovarian folliculogenesis causes infertility in mice. *Development* 146, dev176701. 10.1242/dev.176701 31704792 PMC6918767

[BIO059130C44] Sahin, U., Ferhi, O., Jeanne, M., Benhenda, S., Berthier, C., Jollivet, F., Niwa-Kawakita, M., Faklaris, O., Setterblad, N., de The, H. et al. (2014). Oxidative stress-induced assembly of PML nuclear bodies controls sumoylation of partner proteins. *J. Cell Biol.* 204, 931-945. 10.1083/jcb.201305148 24637324 PMC3998805

[BIO059130C45] Sahin, U., de The, H. and Lallemand-Breitenbach, V. (2022). Sumoylation in physiology, pathology and therapy. *Cells* 11, 814. 10.3390/cells11050814 35269436 PMC8909597

[BIO059130C46] Saitoh, H. and Hinchey, J. (2000). Functional heterogeneity of small ubiquitin-related protein modifiers SUMO-1 versus SUMO-2/3. *J. Biol. Chem.* 275, 6252-6258. 10.1074/jbc.275.9.6252 10692421

[BIO059130C47] Salsman, J., Rapkin, L. M., Margam, N. N., Duncan, R., Bazett-Jones, D. P. and Dellaire, G. (2017). Myogenic differentiation triggers PML nuclear body loss and DAXX relocalization to chromocentres. *Cell Death Dis.* 8, e2724. 10.1038/cddis.2017.151 28358373 PMC5386546

[BIO059130C48] Seifert, A., Schofield, P., Barton, G. J. and Hay, R. T. (2015). Proteotoxic stress reprograms the chromatin landscape of SUMO modification. *Sci. Signal* 8, rs7. 10.1126/scisignal.aaa2213 26152697 PMC6707813

[BIO059130C49] Udagawa, O. and Hirano, S. (2022). How arsenic, an inorganic pollutant, is involved in the physiology of biomolecular condensates in the cell. *Front. Environ. Chem.* 3, 797966. 10.3389/fenvc.2022.797966

[BIO059130C50] Uozumi, N., Matsumoto, H. and Saitoh, H. (2016). Detection of O-propargyl-puromycin with SUMO and ubiquitin by click chemistry at PML-nuclear bodies during abortive proteasome activities. *Biochem. Biophys. Res. Commun.* 474, 247-251. 10.1016/j.bbrc.2016.03.155 27125456

[BIO059130C51] Wang, Z. G., Delva, L., Gaboli, M., Rivi, R., Giorgio, M., Cordon-Cardo, C., Grosveld, F. and Pandolfi, P. P. (1998). Role of PML in cell growth and the retinoic acid pathway. *Science* 279, 1547-1551. 10.1126/science.279.5356.1547 9488655

[BIO059130C52] Wang, Z. B., Ou, X. H., Tong, J. S., Li, S., Wei, L., Ouyang, Y. C., Hou, Y., Schatten, H. and Sun, Q. Y. (2010). The SUMO pathway functions in mouse oocyte maturation. *Cell Cycle* 9, 2640-2646. 10.4161/cc.9.13.12120 20543581 PMC3322456

[BIO059130C53] Wang, L., Wansleeben, C., Zhao, S., Miao, P., Paschen, W. and Yang, W. (2014). SUMO2 is essential while SUMO3 is dispensable for mouse embryonic development. *EMBO Rep.* 15, 878-885. 10.15252/embr.201438534 24891386 PMC4197045

[BIO059130C54] Yamazaki, T., Yamamoto, T., Yoshino, H., Souquere, S., Nakagawa, S., Pierron, G. and Hirose, T. (2021). Paraspeckles are constructed as block copolymer micelles. *EMBO J.* 40, e107270. 10.15252/embj.2020107270 33885174 PMC8204865

[BIO059130C55] Yuan, Y. F., Zhai, R., Liu, X. M., Khan, H. A., Zhen, Y. H. and Huo, L. J. (2014). SUMO-1 plays crucial roles for spindle organization, chromosome congression, and chromosome segregation during mouse oocyte meiotic maturation. *Mol. Reprod Dev.* 81, 712-724. 10.1002/mrd.22339 25123474

