# Supplementary material for: Promyelocytic leukemia nuclear body-like structures can assemble in mouse oocytes
Source: Biol Open. 2022 Jun 6;11(6):bio059130. doi: 10.1242/bio.059130 (PMC9194678; doi:10.1242/bio.059130)
Supplement: Supplementary information [file biolopen-11-059130-s1.pdf]

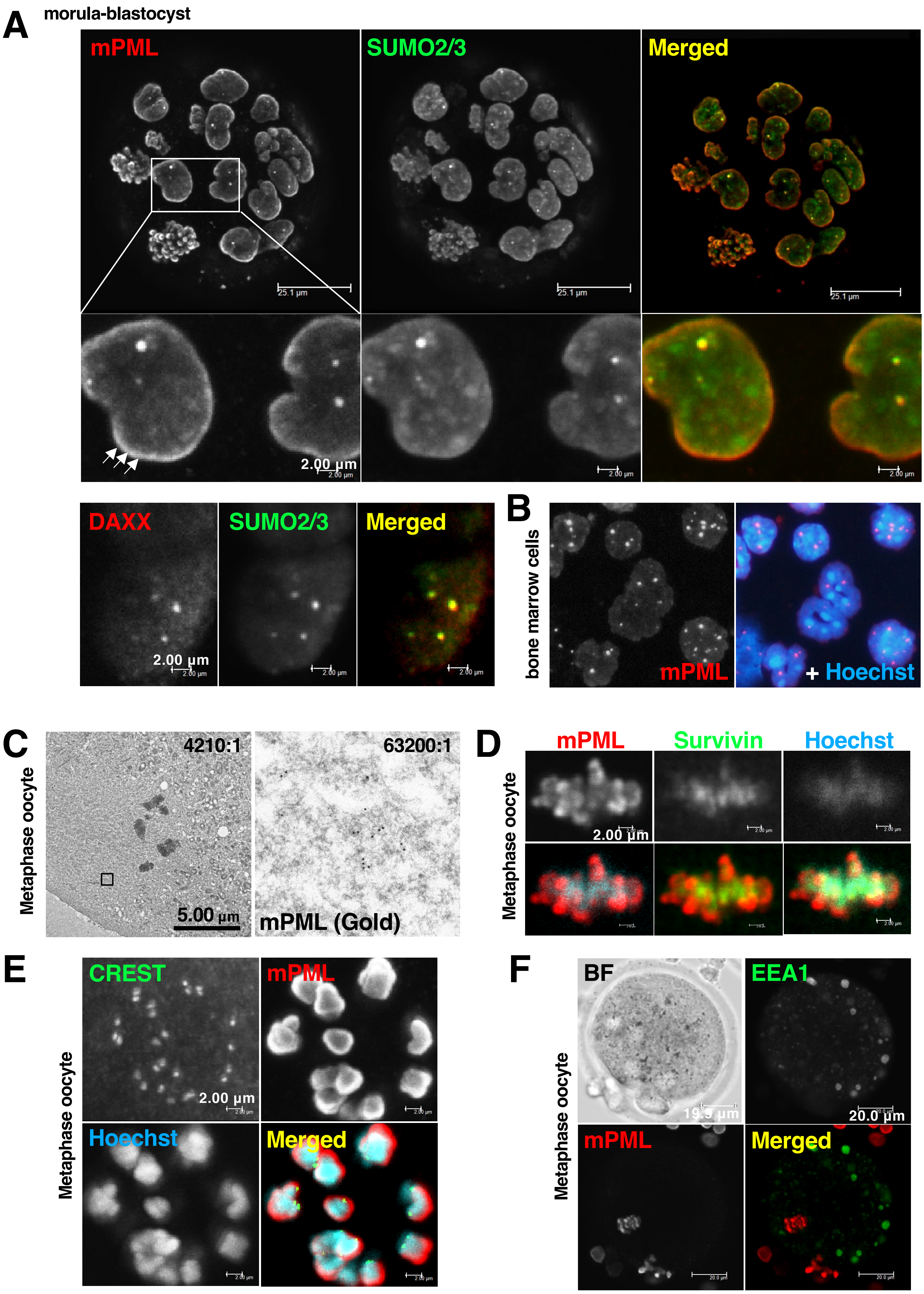


Fig. S1

Biology Open • Supplementary information

**Fig. S1. related to Fig. 1. Subcellular localization of endogenous PML.** (A) (Upper panel) Subcellular localization of endogenous PML (mPML, red) and SUMO2/3 (green) in morula-blastocyst. Arrows in the inset indicate slight enrichment of PML protein beneath the nuclear membrane. (Lower panel) Subcellular localization of DAXX (red) and SUMO2/3 (green). Images were reconstructed as z-stack images. Scale bars, 2.00 μm.

(B) Representative image of endogenous PML-NBs (red) in bone marrow-derived cells. (C) Representative electron micrograph image of peri-chromosomal region in the metaphase oocyte obtained by the culture of germinal vesicle (GV) oocytes for 20 hr. Endogenous PML protein was visualized with primary anti-mouse PML (mPML) antibody and gold particle-conjugated (Gold) secondary antibody. (D–F) Representative fluorescent images of oocytes collected from the ovaries of adult mice. The chromosome arm (D, z-stack), the kinetochore (E, z-stack), and the early endosome of the metaphase oocytes (F) were labeled with anti-survivin antibody, CREST autoantibody, and anti-EEA1 antibody, respectively. BF, bright-field image of the oocyte.

Biology Open • Supplementary information

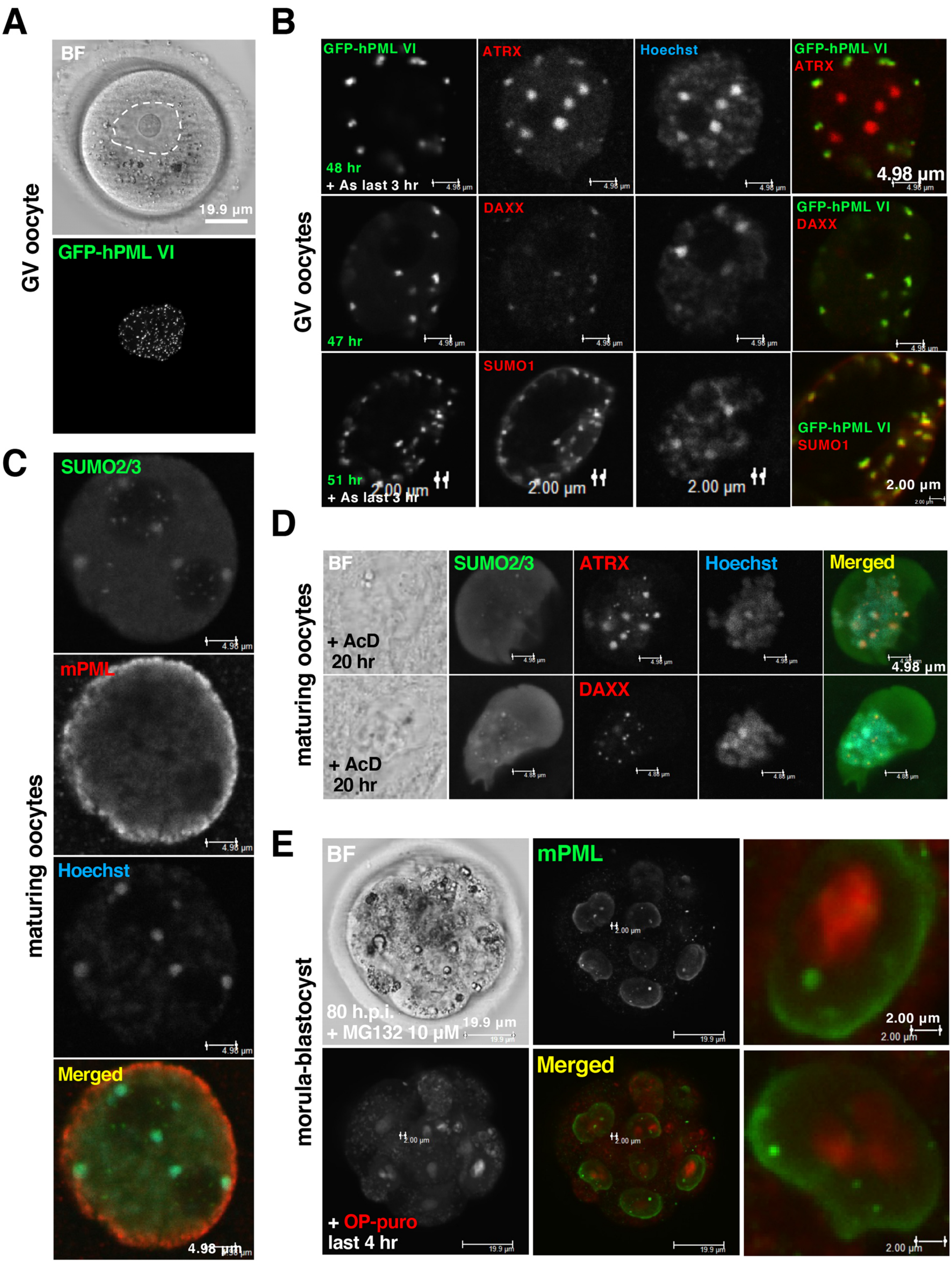


**Fig. S2**

Biology Open • Supplementary information

**Fig. S2. related to Fig. 2. Characterization of nuclear bodies (NBs) deliberately assembled in the nuclei of oocytes.** (A) Representative z-stack image of exogenously formed PML-NBs in the nucleus of the germinal vesicle (GV) oocyte, a model to gain insight into the benefit of a NB-free intranuclear milieu in oocytes. GV oocytes were injected with a plasmid encoding GFP-hPMLVI and cultured for 53 hr. BF, bright-field image of the oocyte. Scale bars, 19.9 µm. (B) Representative images of exogenously formed GFP-hPMLVI-NBs and of representative PML clients in the nuclei of GV oocytes cultured as indicated. Clients were visualized by immunofluorescent staining with anti-ATRX, DAXX, and SUMO1 antibodies. Scale bars, 2.49 and 1.84 µm, respectively. (C) Representative image of the nucleus of maturing oocytes left untreated for 1 hr. Oocytes were stained with anti-mouse PML (mPML, red) and Alexa 488-conjugated anti-SUMO2/3 antibody. Scale bars, 4.98 µm. (D) Representative image of the nucleus of maturing oocytes treated with 1 µM actinomycin D (AcD), a transcriptional inhibitor, for 20 hr. Oocytes were stained with anti-SUMO2/3 antibody and anti-ATRX or DAXX, antibodies. (E) An examination of whether endogenously appearing PML-NBs act as overflow compartments for misfolded proteins. (Left) Representative image of morula-blastocysts at 80 hr post-insemination (80 h.p.i.). From 76 h.p.i., embryos were labeled of newly synthesized aberrant polypeptides with 20 µM OP-puro (red) in the presence of 10 µM MG132 for 4 hr. Endogenously appearing PML-NBs in the nucleus of each blastomere were visualized by immunofluorescent staining with anti-mPML (green) antibody. BF, bright-field image of the embryo. Scale bars, 19.9 µm. (Right) Enlarged images of the nuclei of representative blastomeres. Scale bars, 2.00 µm.

Biology Open • Supplementary information

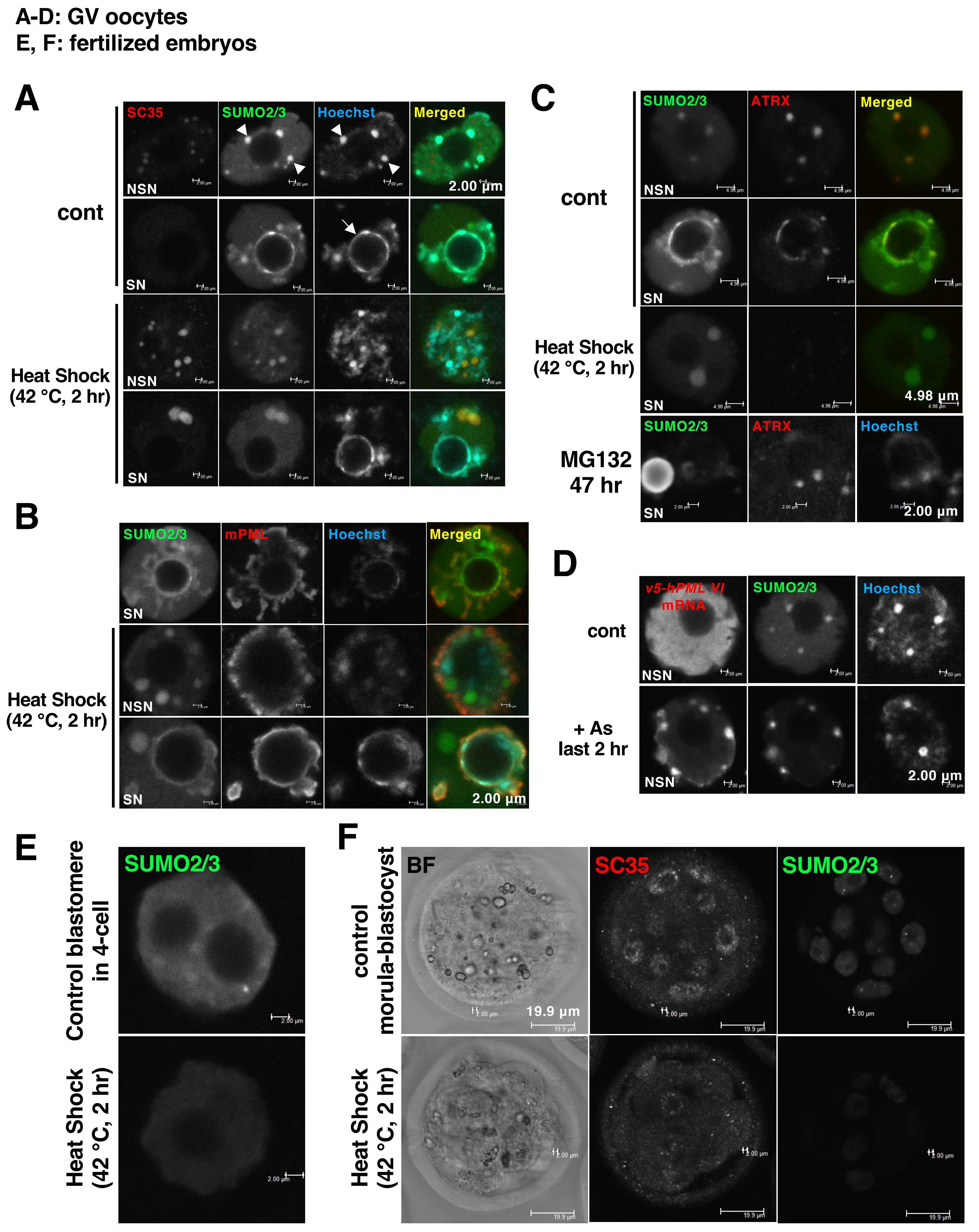


Fig. S3

Biology Open • Supplementary information

**Fig. S3. related to Fig. 3. Characterization of the co-localization of SUMO with enlarged SC35-positive compartments upon proteotoxic stress in oocytes.** (A) Representative images of the nuclei of GV oocytes cultured for 17 hr and then left untreated (cont) or subjected to heat shock at 42 ºC for 2 hr. NSN; non-surrounded nucleolus stage, SN; surrounded nucleolus stage. Arrowheads indicate SUMO deposition at sites of condensed DNA (peri-centromeric heterochromatin) in NSN-oocytes. Arrow indicates the heterochromatin rim in SN-oocytes. Oocytes were stained with anti- SC35 (red) and Alexa 488-conjugated anti-SUMO2/3 (green) antibodies. Scale bars, 2.00 µm. (B) Representative images of the nuclei of germinal vesicle (GV) oocytes treated with dimethyl sulfoxide (DMSO, vehicle control) for 24 hr or subjected to heat shock at 42 ºC for 2 hr. Endogenous PML was visualized by staining with anti-mouse PML (mPML, red) antibody. The oocytes were further stained with Alexa 488-conjugated anti-SUMO2/3 (green) antibody. (C) Representative images of the nuclei of GV oocytes cultured for 1 hr and then left untreated (cont) or subjected to heat shock at 42 ºC for 2 hr, or GV oocytes treated with 10 µM MG132 for 47 hr. Oocytes were stained with anti-ATRX (red) and Alexa 488-conjugated anti-SUMO2/3 (green) antibodies. (D) Representative image of the nucleus of GV oocytes injected with the mRNA transcribed *in vitro* from plasmid v5-hPMLVI (encoding wild-type human PMLVI) and cultured for 46 hr then left untreated (cont) or subjected to treatment with 3 µM arsenite for 2 hr. Oocytes were stained with anti-human PML (red) and Alexa 488-conjugated anti-SUMO2/3 (green) antibodies. Scale bars, 2.00 µm. (E) Representative image of the nucleus of the blastomere in 4-cell embryos. Embryos at 48 hr post-insemination (48 h.p.i.) that subsequently were left untreated (control) or exposed to heat shock at 42 ºC for 2 hr. Embryos were stained with Alexa

Biology Open • Supplementary information

488-conjugated anti-SUMO2/3 antibody. Control blastomere corresponds to that containing PML-NB shown in Fig. 1B. (F) Representative image of morula-blastocysts at 80 h.p.i. that subsequently were left untreated (cont) or exposed to heat shock at 42 ºC for 2 hr. Embryos were stained with anti-SC35 (red) and Alexa 488-conjugated anti-SUMO2/3 (green) antibodies. BF, bright-field images of the embryos. Scale bars, 19.9 µm.

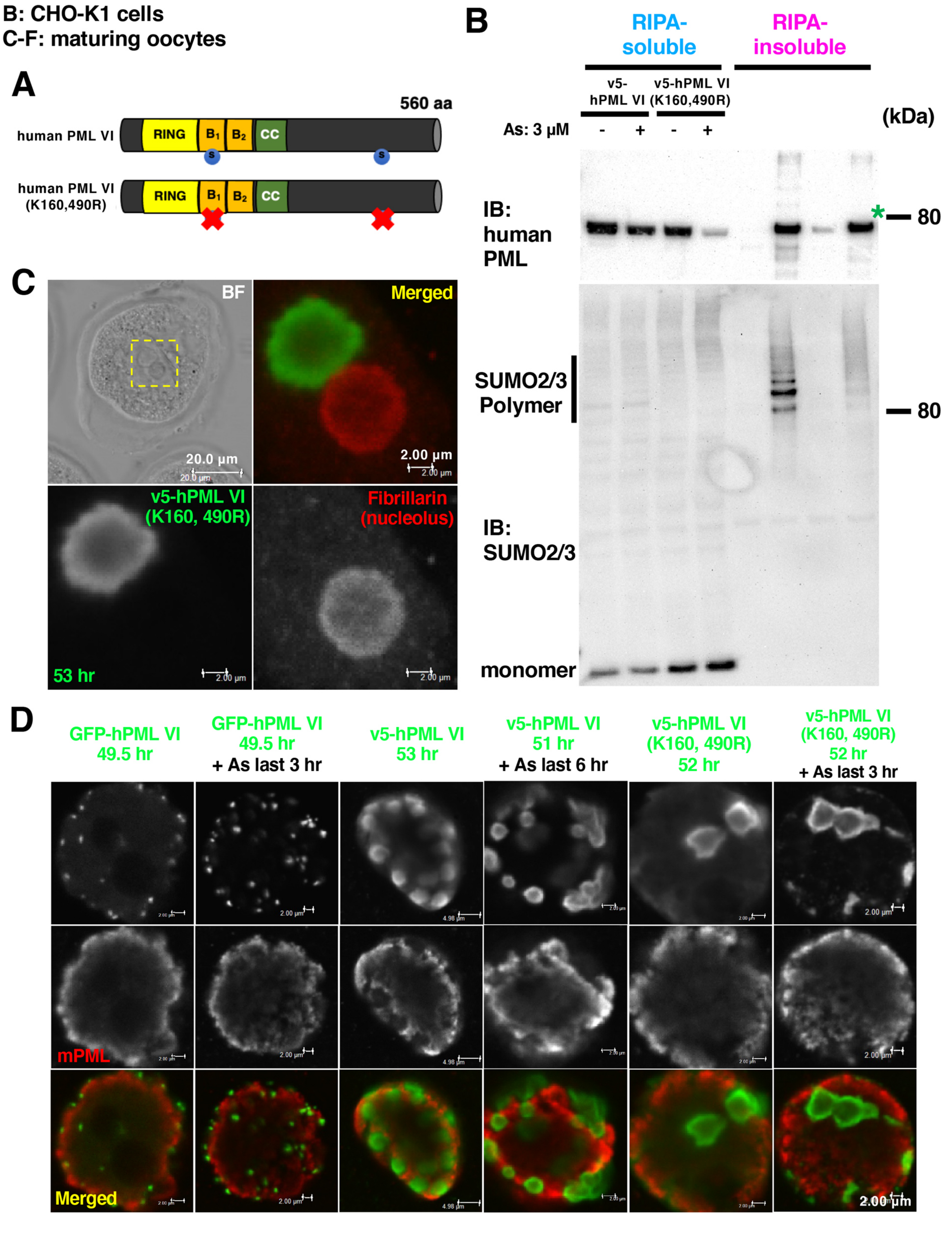


Fig. S4 (Continued)

**All photos shown in this figure were taken from maturing oocytes**

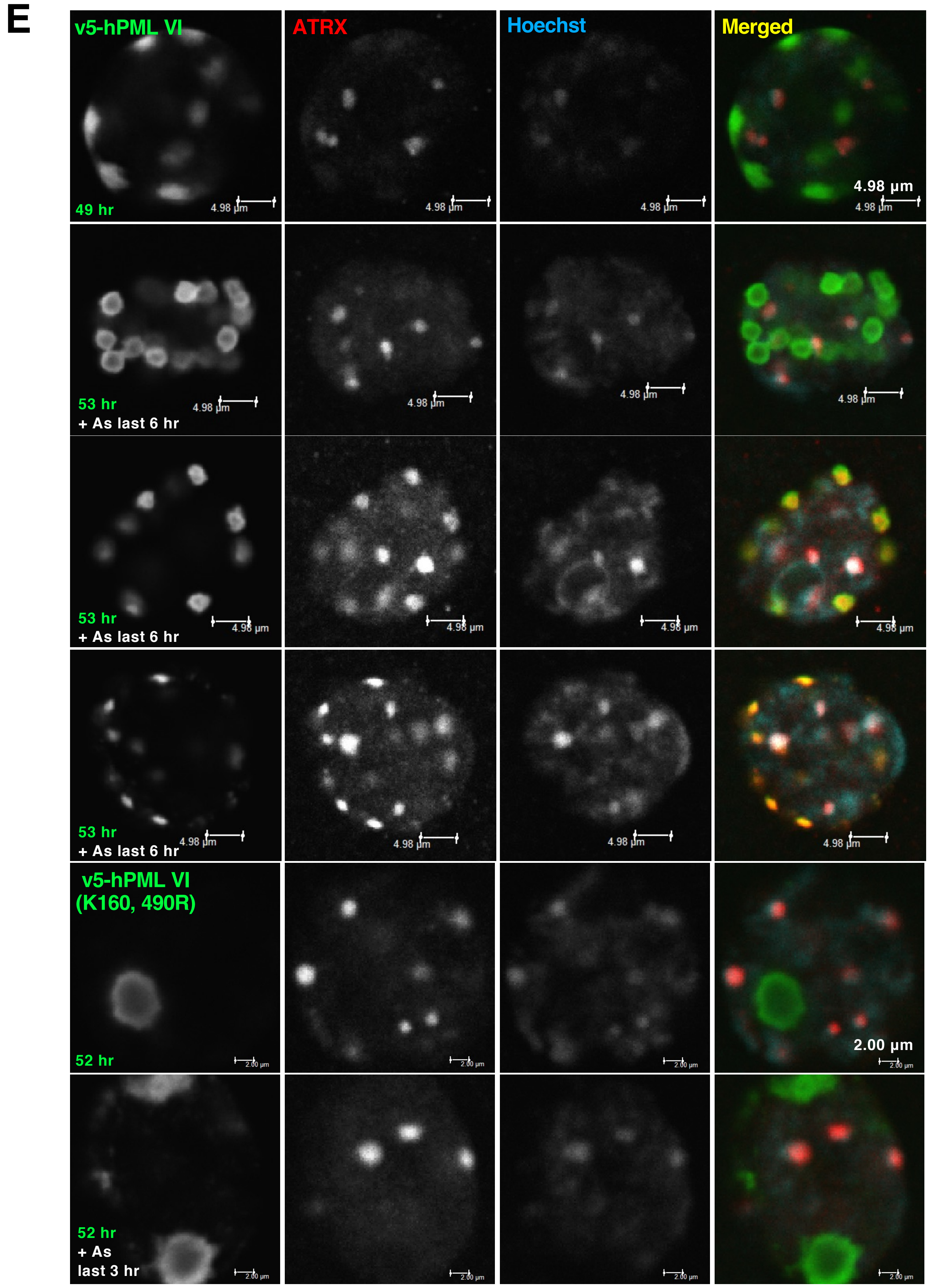


**Fig. S4 (continued)**

Biology Open • Supplementary information

All photos shown in this figure were taken from maturing oocytes

F

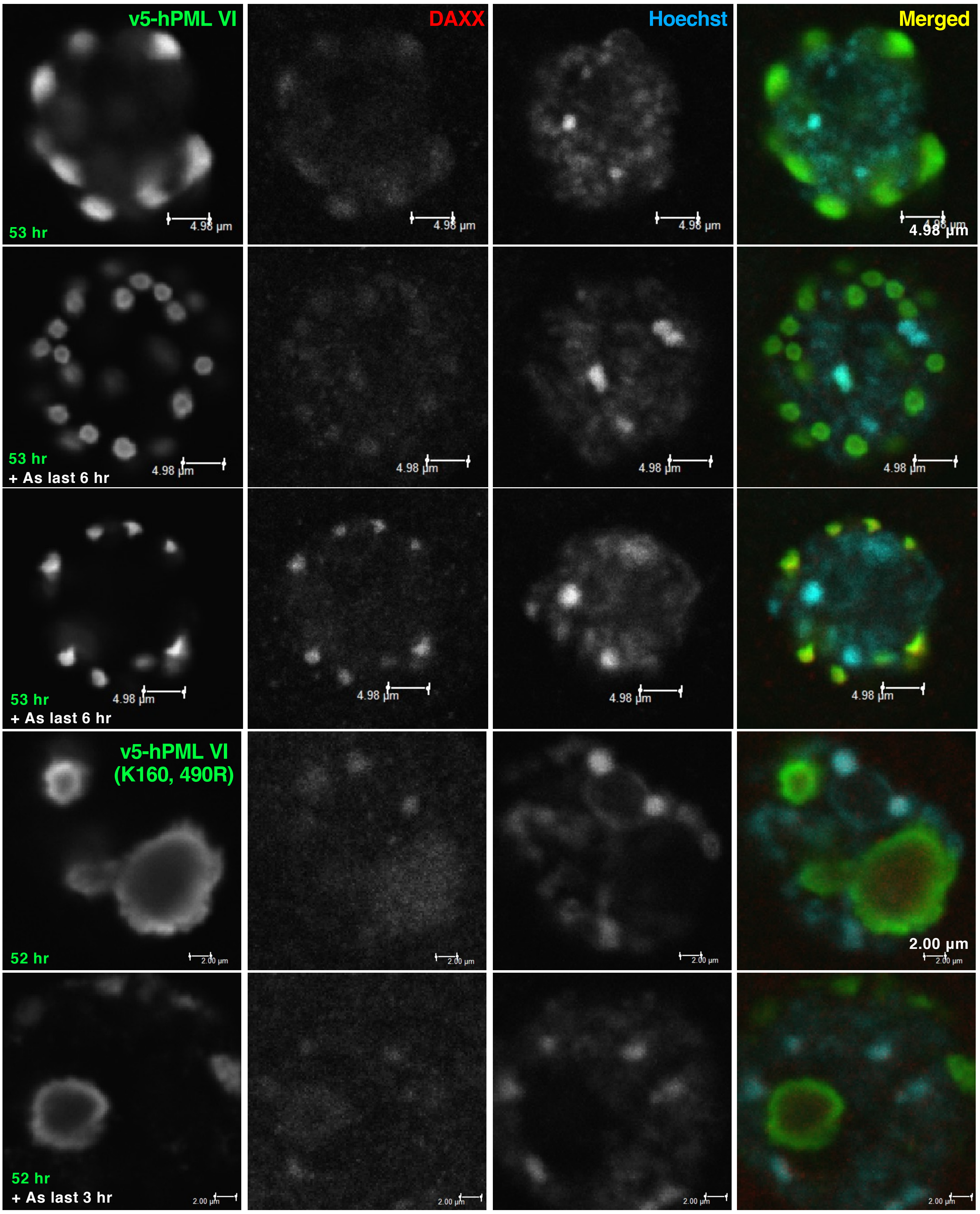


Fig. S4

**Fig. S4. related to Fig. 4. Preparations for modifying the SUMO availability in oocytes.** (A) Schematic domain structures of wild-type human PMLVI and a mutant version of the same protein that has limited avidity for SUMO; these proteins were encoded by the plasmids v5-hPMLVI and v5-hPMLVI (K160, 490R), respectively. RING: Really Interesting New Gene domain, with conserved cysteine. B1 and B2: zinc-binding boxes. CC: coiled-coil domain. Two SUMOylation sites are indicated by aqua blue circles labeled with an S. These sites were mutated from lysine to arginine in the SUMOylation-deficient mutant protein encoded by the v5-hPMLVI (K160, 490R) construct. (B) Expression of constructs and biochemical response of their products to arsenite. Immunoblot analysis of CHO-K1 cells transiently expressing wild-type human PMLVI or the SUMOylation-deficient mutant protein. The fractions soluble or insoluble to radioimmunoprecipitation (RIPA) lysis buffer were immunoblotted (IB:) with the indicated antibodies. The cells were exposed to 3 µM arsenite (As) for 3 hr. Green asterisk indicates the biochemical response of the v5-hPMLVI (K160, 490R) -encoded mutant protein to arsenite. The biochemical response was comparable to that seen with the unmutated protein encoded by the v5-hPMLVI construct. (C) Representative image of the nucleus of maturing oocytes injected with plasmid v5-hPMLVI (K160, 490R) and cultured for 53 hr. Oocytes were stained with anti-human PML (green) and Fibrillarin (red) antibodies. The area enlarged is shown in the bright-field (BF) image. Scale bars, 2.00 µm. (D) Representative images of the nuclei of maturing oocytes injected with plasmid encoding GFP-hPMLVI, with plasmid v5-hPMLVI or v5-hPMLVI (K160, 490R) followed by the culture as indicated. Oocytes were stained with anti-mouse PML (mPML, red) antibody. Except for oocytes injected with GFP-hPMLVI encoding plasmid, oocytes were stained with anti-human PML (green) antibody. Scale bars, 2.00 µm. (E and F) Representative images of the nuclei of maturing oocytes injected with plasmid v5-hPMLVI or v5-hPMLVI (K160, 490R) followed by the culture as indicated. Oocytes were stained with anti-human PML (green) antibody and anti-ATRX (E, red), or DAXX (F, red) antibodies.

Biology Open • Supplementary information
